# Comprehensive proteogenomic characterization of rare kidney tumors

**DOI:** 10.1016/j.xcrm.2024.101547

**Published:** 2024-05-03

**Authors:** Ginny Xiaohe Li, Lijun Chen, Yi Hsiao, Rahul Mannan, Yuping Zhang, Jie Luo, Francesca Petralia, Hanbyul Cho, Noshad Hosseini, Felipe da Veiga Leprevost, Anna Calinawan, Yize Li, Shankara Anand, Aniket Dagar, Yifat Geffen, Chandan Kumar-Sinha, Seema Chugh, Anne Le, Sean Ponce, Shenghao Guo, Cissy Zhang, Michael Schnaubelt, Nataly Naser Al Deen, Feng Chen, Wagma Caravan, Andrew Houston, Alex Hopkins, Chelsea J. Newton, Xiaoming Wang, Daniel A. Polasky, Sarah Haynes, Fengchao Yu, Xiaojun Jing, Siqi Chen, Ana I. Robles, Mehdi Mesri, Mathangi Thiagarajan, Eunkyung An, Gad A. Getz, W. Marston Linehan, Galen Hostetter, Scott D. Jewell, Daniel W. Chan, Pei Wang, Gilbert S. Omenn, Rohit Mehra, Christopher J. Ricketts, Li Ding, Arul M. Chinnaiyan, Marcin P. Cieslik, Saravana M. Dhanasekaran, Hui Zhang, Alexey I. Nesvizhskii, Clinical Proteomic Tumor Analysis Consortium, Alexander J. Lazar, Amanda G. Paulovich, Andrzej Antczak, Anthony Green, Avi Ma’ayan, Barb Pruetz, Bing Zhang, Boris Reva, Brian J. Druker, Charles A. Goldthwaite, Chet Birger, D.R. Mani, David Chesla, David Fenyö, Eric E. Schadt, George Wilson, Iga Kołodziejczak, Ivy John, Jason Hafron, Josh Vo, Kakhaber Zaalishvili, Karen A. Ketchum, Karin D. Rodland, Kristen Nyce, Maciej Wiznerowicz, Marcin J. Domagalski, Meenakshi Anurag, Melissa Borucki, Michael A. Gillette, Michael J. Birrer, Nathan J. Edwards, Negin Vatanian, Pamela VanderKolk, Peter B. McGarvey, Rajiv Dhir, Ratna R. Thangudu, Reese Crispen, Richard D. Smith, Samuel H. Payne, Sandra Cottingham, Shuang Cai, Steven A. Carr, Tao Liu, Toan Le, Weiping Ma, Xu Zhang, Yin Lu, Yvonne Shutack, Zhen Zhang

**Affiliations:** 1 Department of Pathology, University of Michigan, Ann Arbor, MI 48109, USA; 2 Department of Pathology, Johns Hopkins University School of Medicine, Baltimore, MD 21231, USA; 3 Department of Computational Medicine and Bioinformatics, University of Michigan, Ann Arbor, MI 48109, USA; 4 Michigan Center for Translational Pathology, Department of Pathology, University of Michigan, Ann Arbor, MI 48109, USA; 5 Department of Genetics and Genomic Sciences, Icahn School of Medicine at Mount Sinai, New York, NY 10029, USA; 6 Department of Medicine, Washington University in St. Louis, St. Louis, MO 63110, USA; 7 McDonnell Genome Institute, Washington University in St. Louis, St. Louis, MO 63108, USA; 8 Broad Institute of Massachusetts Institute of Technology and Harvard, Cambridge, MA 02142, USA; 9 Cancer Center and Department of Pathology, Massachusetts General Hospital, Boston, MA 02115, USA; 10 Department of Chemical and Biomolecular Engineering, Johns Hopkins University Whiting School of Engineering, Baltimore, MD 21218, USA; 11 Department of Oncology, Johns Hopkins University School of Medicine, Baltimore, MD 21287, USA; 12 Department of Biomedical Engineering, Johns Hopkins University Whiting School of Engineering, Baltimore, MD 21218, USA; 13 Van Andel Research Institute, Grand Rapids, MI 49503, USA; 14 Office of Cancer Clinical Proteomics Research, National Cancer Institute, Rockville, MD 20850, USA; 15 Frederick National Laboratory for Cancer Research, Frederick, MD 21702, USA; 16 Urologic Oncology Branch, Center for Cancer Research, National Cancer Institute, National Institutes of Health, Bethesda, MD 20892, USA; 17 Department of Urology, Johns Hopkins University School of Medicine, Baltimore, MD 21287, USA; 18 Department of Internal Medicine, Human Genetics, and School of Public Health, University of Michigan, Ann Arbor, MI 48109, USA; 19 Department of Genetics, Washington University in St. Louis, St. Louis, MO 63130, USA; 20 Siteman Cancer Center, Washington University in St. Louis, St. Louis, MO 63130, USA; 21 Howard Hughes Medical Institute, University of Michigan, Ann Arbor, MI 48109, USA; 22 Rogel Cancer Center, University of Michigan, Ann Arbor, MI 48109, USA; 23 Department of Urology, University of Michigan, Ann Arbor, MI 48109, USA

**Keywords:** non-clear cell renal cell carcinoma, weighted genome instability index, cell-of-origin, proteogenomics, differential diagnosis biomarkers, prognostic marker, metabolomics, CPTAC, phosphoproteomics, glycoproteomics

## Abstract

Non-clear cell renal cell carcinomas (non-ccRCCs) encompass diverse malignant and benign tumors. Refinement of differential diagnosis biomarkers, markers for early prognosis of aggressive disease, and therapeutic targets to complement immunotherapy are current clinical needs. Multi-omics analyses of 48 non-ccRCCs compared with 103 ccRCCs reveal proteogenomic, phosphorylation, glycosylation, and metabolic aberrations in RCC subtypes. RCCs with high genome instability display overexpression of IGF2BP3 and PYCR1. Integration of single-cell and bulk transcriptome data predicts diverse cell-of-origin and clarifies RCC subtype-specific proteogenomic signatures. Expression of biomarkers MAPRE3, ADGRF5, and GPNMB differentiates renal oncocytoma from chromophobe RCC, and PIGR and SOSTDC1 distinguish papillary RCC from MTSCC. This study expands our knowledge of proteogenomic signatures, biomarkers, and potential therapeutic targets in non-ccRCC.

## Highlights

• Subtype-specific features across a pan-RCC cohort revealed by multi-omics data resources
• Diverse cell-of-origin predictions and tumor signatures revealed by snRNA-seq
• Proteogenomic, metabolic, glycoproteomic signatures of high-wGII tumors
• Biomarkers GPNMB, ADGRF5, MAPRE3 for chRCC/RO and PIGR, SOSTDC1 for pRCC

## Graphical abstract

**Figure:**
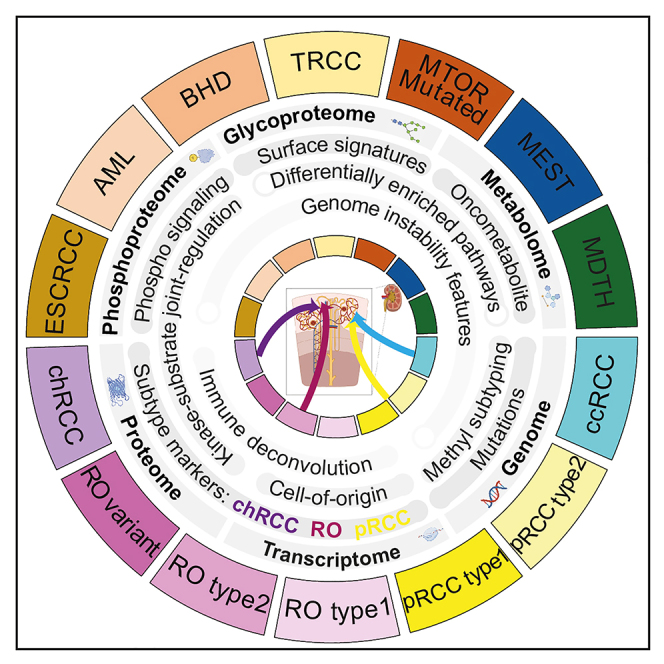

## Teaser

Li et al. perform comprehensive multi-omics characterization of a broad range of renal cell carcinomas, improving our understanding of the biology, proteogenomics, post-translational modifications, and metabolism of kidney cancer. These findings identify important biomarkers IGF2BP3, PYCR1, GPNMB, ADGRF5, MAPRE3, PIGR, and SOSTDC1 that illuminate molecular differences within renal cell carcinoma.

## Introduction

World Health Organization (WHO) 2022 lists 20 different renal cell carcinoma (RCC) subtypes, of which 7 are defined by specific molecular aberrations.^1^ Non-clear cell RCC (ccRCC) accounts for ∼20% of RCCs and encompasses a variety of rare subtypes largely defined by histopathologic features,^1^^,^^2^^,^^3^ collectively referred to here as non-ccRCCs. Among non-ccRCC tumors, papillary RCC (pRCC) (10%–15%) and chromophobe RCC (chRCC) (3%–5%) are relatively common, while the other subtypes are much rarer. Several rare renal tumors with benign clinical courses show morphological overlap with malignant counterparts.^4^^,^^5^ We have previously discovered several biomarkers to aid in differential diagnosis of many RCC subtypes^6^^,^^7^^,^^8^^,^^9^; however, diagnosis in limited biopsy samples settings remains challenging.^10^^,^^11^ In addition, biomarkers to identify patients at high risk of disease relapse within each RCC subtype who will benefit from increased surveillance and adjuvant therapy is another unmet clinical need.^12^ Our recent ccRCC study^13^ nominated biomarkers associated with features of worse prognosis, such as genome instability (GI). Similar markers for non-ccRCC tumors remain to be identified. Similarly, while immune checkpoint and angiogenesis inhibitors are treatment options in metastatic ccRCC,^14^^,^^15^^,^^16^ immune infiltration and tumor vascularity vary widely among non-ccRCC tumors, necessitating further evaluation of markers of responsiveness.

To address the knowledge gaps in non-ccRCC differential diagnoses, prognoses, and therapeutic avenues, as part of the Clinical Proteomic Tumor Analysis Consortium (CPTAC), we performed integrated proteogenomic multi-omic analysis of non-ccRCC and ccRCC tumors. Besides the few studies detailing genomics,^17^^,^^18^^,^^19^ transcriptomics,^17^^,^^19^ and proteomics^20^^,^^21^^,^^22^^,^^23^ of rare RCCs, multi-omic profiling is largely unavailable. Here, we report multi-omic analysis of 48 non-ccRCC cases (non-ccRCC cohort) along with the reported ccRCC (n = 103) discovery cohort samples.^24^ This integrative pan-RCC analysis identified shared and subtype-specific proteogenomic, glycoproteomic, metabolic features across RCC subtypes, nominated various diagnostic biomarkers, and provided validation for selected candidates. Single-nucleus RNA sequencing (snRNA-seq) analysis captured transcriptomic heterogeneity of tumor subclusters and helped predict cell-of-origin. Combined, the data from this study provide a rich resource for identifying diagnostic biomarkers, disease mechanisms, and potentially new therapeutic targets for non-ccRCC subtypes.

## Results

### Specimens and multi-omics data types

We performed multi-omics data analysis of 48 non-ccRCC and 103 ccRCC tumors and 101 normal adjacent tissues (NATs) (22 and 79 from non-ccRCC and ccRCC patients, respectively) (Figure S1A; Table S1). Multi-omics data available from common sample aliquots^25^ include whole-genome sequencing, whole-exome sequencing, DNA methylation profiling, and RNA-seq for all 151 tumor samples, and RNA-seq data for 89 NATs (ccRCC n = 71; non-ccRCC n = 18) (Figure S1A). snRNA-seq data were generated for eight non-ccRCC tumors.

**Figure 1:**
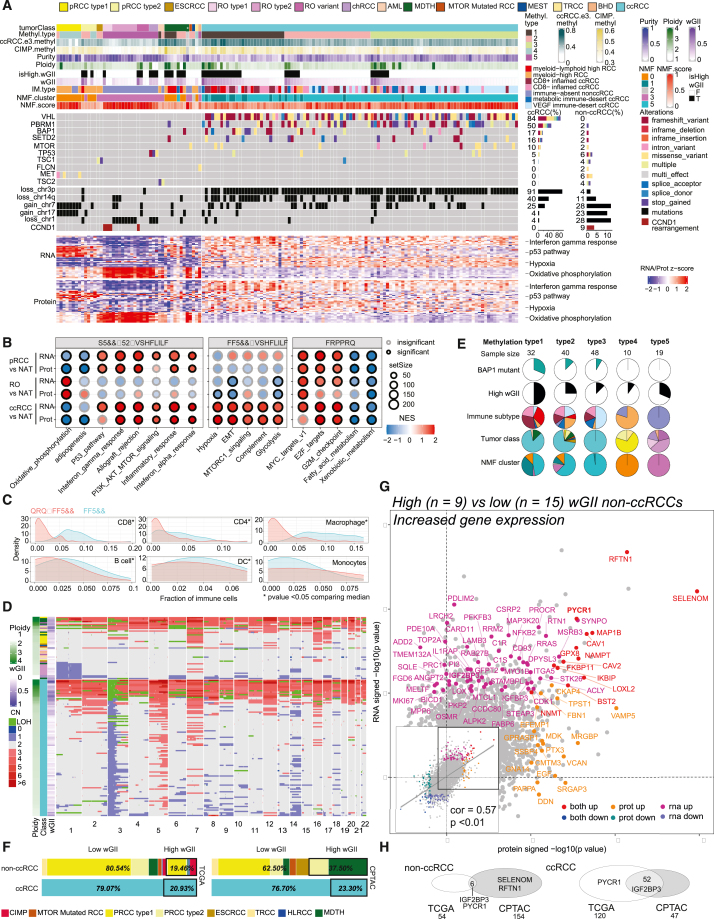
Proteogenomic biomarkers of copy number-based genome instability in renal cell carcinoma (A) Proteogenomic aberration landscape of ccRCC and non-ccRCC. Top panel: histo-molecular annotations condensed as tracks (∗excluded sample). RNA and protein automatic relevance determination in non-negative matrix factorization (ARD-NMF) classification. Middle panel: non-ccRCC display distinct recurrent events. Bottom panel: heatmaps show the top 10 differentially expressed genes and proteins enriched in annotated biological processes. Top 20 protein and RNA features (log2 fold change) from selected pathways. (B) Differentially enriched pathways (RNA and protein) among the various RCC subtypes. (C) Predicted immune composition for ccRCC and non-ccRCC. (D) Heatmap of absolute copy number variation (CNV) deduced from CNVEX output for non-ccRCC (top) and ccRCC (bottom) sorted by ploidy. Ploidy, RCC subtype, wGII annotations tracks provided (left). (E) Distribution of BAP1 mutation, wGII, immune subtype, tumor classes, and NMF clustering in five methylation subgroups. Significant enrichment (p < 0.01) of BAP1 mutation, high wGII, myeloid-lymphoid high immune subtype, and NMF cluster1 hyper-methylated group. (F) Subtype composition among low- and high-wGII tumors, in TCGA (left) and CPTAC (right) non-ccRCC (upper), and ccRCC (lower) cohorts. Bold black borders, high-wGII samples. (G) Comparison of significance levels (signed –log10 p value) between protein (x axis) and mRNA (y axis) under high to low-wGII comparison within a subset of non-ccRCC samples. Significantly upregulated genes are labeled and colored. The inset shows the global correlation between the changes. (H) Overlap between TCGA and CPTAC high-wGII mRNA expression gene markers in non-ccRCC (left) and ccRCC (right).

Histopathological subtyping information and signature molecular aberrations such as copy number variation patterns, somatic/germline mutations, marker gene expression, and gene fusions were collectively assessed to arrive at tumor-molecular annotation (Figure 1A; Table S1).^26^ Based on the WHO 2018 renal tumor histological classification (available at data freeze), the analysis cohort comprises 103 ccRCC, 15 renal oncocytomas (ROs), 13 pRCC (8 of them with type 1 features; WHO 2018), 3 chRCC, 2 angiomyolipoma (AML), 2 eosinophilic solid and cystic RCC (ESCRCC), 1 Birt-Hogg-Dube syndrome-associated renal cell carcinoma, 1 mixed epithelial and stromal tumor of the kidney, 1 MTOR mutated RCC, 1 translocation RCC (TRCC), and 8 tumors where genomics aberrations patterns did not concur with histological classification were annotated as molecularly divergent to histology (MDTH) (Figure 1A; Table S1).

The sample cohorts have comparable demographic and clinical composition except for a higher proportion of female patients in non-ccRCC compared with ccRCC cohorts (p = 0.036) (Figure S1B). The multi-omics dataset can be queried using the interactive ProTrack website http://ccrcc-conf.cptac-data-view.org (Figure S1B).^27^ We identified a total of 12,299 proteins, 9,396 phosphorylated proteins, and 1,035 glycoproteins, of which 9,528 proteins, 6,465 phosphorylated proteins, and 639 glycoproteins were quantified in more than half of all samples. Principal-component analysis (PCA) of global proteome, phosphoproteome, and glycoproteome data showed clear separation between different tumor subtypes and normal samples in two-dimensional space (Figure S1C). Known mutation consequences on kinase protein expression and phosphorylation are consistent with previous findings^18^^,^^28^ (Figures S1D, S3B, and S3C).

### Subtype-specific proteogenomic signatures

Different subtypes of non-ccRCC tumors displayed recurrent genomic aberrations distinct from ccRCC (Figure 1A). Notable non-ccRCC subtype-specific events include: the signature chromosomal losses and *TP53* mutations in chRCC (3/3 cases), chr7/17 gain (8/13 cases), and *MET* mutations (3/13 cases) in pRCC, *TSC* gene mutations in ESCRCC (2/2 cases) and AML tumors (2/2 cases), and the *TFE3* gene fusion in a TRCC case. Consistent with previous classification of RO molecular subtypes,^18^ RO type 1 was enriched with *CCND1* gene rearrangement with a diploid genome, and type 2 was marked by one copy loss of chromosome 1 (chr1), and RO cases that did not show either of these molecular features were categorized as the “RO variant” subgroup.^18^ Gene set enrichment analysis (GSEA) revealed several interesting pathway similarities and differences among RCC subtypes (Figure 1B; Tables S2 andS3). For instance, immune/inflammatory response concepts, including allograft rejection, inflammatory response, interferon alpha/gamma pathways, were significantly upregulated, especially at the protein levels, in both pRCC and ccRCC. In contrast, glycolysis, hypoxia, and epithelial-to-mesenchymal transition (EMT) were significantly enriched in the ccRCC proteome but showed a negative enrichment trend in pRCC and RO. Interestingly, oxidative phosphorylation showed significant positive enrichment in RO but was down in pRCC and ccRCC as expected (Figures 1A and 1B).^18^^,^^22^^,^^24^^,^^29^

Next, the status of tumor-immune infiltration was assessed through immune deconvolution followed by clustering analysis (STAR Methods; Figure 1A). In addition to four previously described ccRCC clusters,^24^ three non-ccRCC clusters were identified: one myeloid-lymphoid high non-ccRCC cluster, a myeloid-high cluster containing most pRCC samples, and an immune-absent cluster comprising all oncocytic tumors (Figure 1A). Overall, the extent of immune infiltration was lower in non-ccRCC than in ccRCC (Figure 1C). High weighted genome instability (wGII) containing cases were observed among non-ccRCC (∼37%), ccRCC (∼23%) in the CPTAC, and non-ccRCC (∼20%) and ccRCC (21%) in the TCGA cohorts (Figures 1D–1F). Interestingly, myeloid-lymphoid high non-ccRCC tumors showed high immune infiltration and high wGII (Figures 1A and S1E).

### Proteogenomics of high-wGII samples

Integrative analysis of RNA, protein, and phosphorylation site level expression data performed using automatic relevance determination non-negative matrix factorization (ARD-NMF)^30^ defined six multi-omics clusters. Among these, most pRCC tumors clustered in ARD-NMF-0, oncocytic tumors (RO, chRCC) in ARD-NMF-3, ccRCC samples were distributed in ARD-NMF-1 and -5, while the NATs populated ARD-NMF-2 and ARD-NMF-4 (Figure 1A; Table S2). The smaller ARD-NMF-1 is associated with DNA hypermethylation Methyl1 group, higher-grade ccRCC,^13^ and worse prognosis, while the larger ARD-NMF-5 ccRCC cluster is enriched (p < 0.05, chi-square test) in low-grade ccRCC tumors. Next, as DNA hypermethylation subgroups have been associated with worse survival,^17^^,^^19^ we performed consensus clustering with DNA methylation data and identified five different methylation clusters. Methyl3 and Methyl5 were largely subtype specific and contained ccRCC and all oncocytic tumors, respectively. Interestingly, Methyl1 was enriched with ccRCC samples with high wGII, *BAP1* mutants, and a subset of non-ccRCC samples with high wGII and high ploidy mostly from the MDTH category (Figures 1E and S1E**)**.

We next compared the mRNA and protein differential expression (DE) between high-wGII (n = 9) versus low-wGII (n = 15) non-ccRCC samples including pRCCs, TRCC, ESCRCC, MTOR mutated, and MDTH (Figure 1G; Table S4). A collection of prominent wGII markers was concordantly identified including the mitochondrial proline biosynthetic pathway enzyme PYCR1, associated with cancer cell survival, invasion, and progression across multiple cancer types.^31^^,^^32^^,^^33^ PYCR1 was confirmed to be upregulated in high-wGII samples based on RNA *in situ* hybridization (RNA-ISH) (Figure S1F). In addition, the RNA binding protein and N6-methyladenosine reader IGF2BP3 showed a significantly higher mRNA expression in non-ccRCC high-wGII samples (Figure 1G), and upregulation trend noted in protein expression was validated by immunohistochemistry (IHC) (Figure S1G). Importantly, high IGF2BP3 RNA expression was noted in high-wGII samples across CPTAC and TCGA datasets (Figure 1H). IGF2BP3 has been associated with worse survival in several cancer types,^34^ but has not been previously associated with high wGII. In general, minimal overlap of high wGII associated differentially expressed genes and proteins was noted between ccRCC and non-ccRCC, but Hallmark pathways associated with high-wGII cases included cell-cycle/proliferation concepts (e.g., enrichment of E2F targets, G2-M checkpoint), as well as immune- and inflammation-related concepts, EMT, hypoxia, and glycolysis (Figure S1H). Notably, the MDTH non-ccRCC tumors that are largely genome unstable (6/7) tend to be both hypermethylated (4/7) and immune infiltrated (6/7).

### Non-ccRCC snRNA-seq reveals intra-tumor transcriptomic heterogeneity and low immune infiltration

**Figure 2:**
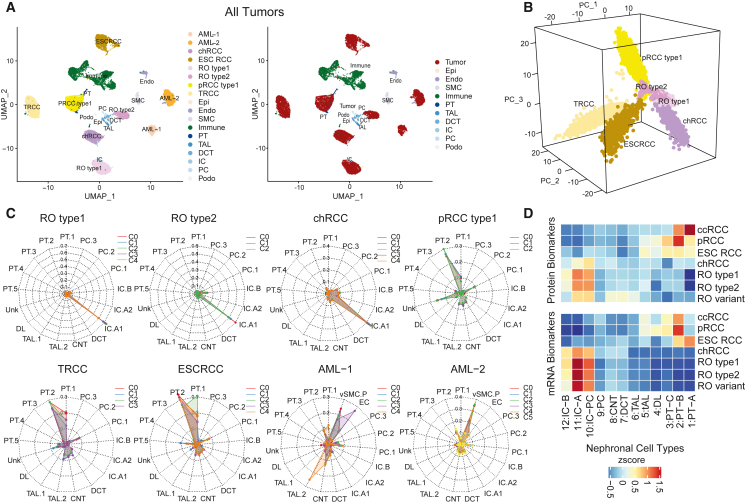
Tumor transcriptomic heterogeneity, immune infiltration status, and tumor cell-of-origin by snRNA-seq (A) UMAP of snRNA-seq data from eight non-ccRCC tumors. Nuclei are colored by RCC subtypes for tumor cells (left) and cell types (right). (B) First three principal components of six tumors (AML excluded) colored by tumor types. (C) Probabilities of cell-of-origin are predicted by a random forest classifier for different tumor subclusters for RCC subtypes. Classifier was trained on Lake et al.^35^ benign renal epithelial cell snRNA-seq data. (D) Averaged abundance of DE protein (top) and mRNA (bottom) markers from each RCC subtype versus NATs among the epithelial cell types identified from normal kidney scRNA-seq data.^36^

To investigate cellular level associations in non-ccRCCs, snRNA-seq data for 8 samples (9,673 single-nuclei transcriptomes [median 10,592 nuclei/sample]), were analyzed along with 3 ccRCC samples from our companion study^13^ (Table S5). Dimensionality reduction analysis post downsampling (2,000 nuclei/sample) showed distinct immune, endothelial, and stromal cell clusters irrespective of the patient of origin (Figures 2A and S2A), while the tumor epithelia formed patient-specific clusters (Figure 2B). Most non-ccRCC samples had higher tumor cell fractions, implying higher tumor content and lower immune infiltration^17^ compared with ccRCC (Figures S2A–S2C) except for the two high immune fraction AML cases. ROs and chRCC were closer in space, while pRCC, ESCRCC, and TRCC were more distinctly positioned.

Multiple tumor subclusters in AML samples revealing intra-tumor transcriptomic heterogeneity were associated with different constituent tumor cell types, presumably a result of trans-differentiation from a common cell of origin^37^ (Figure S2D). AML tumor compartment comprises an admixture of cells that are histologically and molecularly similar to vascular (angio-), smooth muscle (myo-), and fat (lipo-) lineages.^38^ Finally, among the ROs, RO type 1 showed multiple tumor subclusters, including one entirely associated with the S-phase of the cell cycle, indicating higher tumor proliferation rates (Figure S2E). Among other tumors analyzed, all tumor clusters from a given sample showed corresponding mRNA expression changes associated with clonal copy number events such as chr7 and 17 gains (pRCC) and chr1 loss (type 2 RO) (Figure S2F).

Tumor single-cell transcriptome data have been employed to predict cell-of-origin of tumors, using a random forest model trained on benign nephronal epithelial cell types.^36^ We performed similar analysis using snRNA-seq datasets from various RCC subtypes and the publicly available benign human kidney samples^35^ (Figure 2C; STAR Methods). Interestingly, TRCC, ESCRCC, and pRCC showed highest origin-probability to the proximal tubule 2 (PT2) population, a rare cell type that is equivalent to the PT-B population (designated from single-cell RNA-seq data) that we previously demonstrated to contain stem-like marker gene expression^36^ (Figure 2D). In contrast, the ROs and chRCC consistently showed highest probability to the intercalated-A (IC-A) population, suggesting a distal nephron origin (Figure 2D). Among the AML tumor compartments, we noted similarities between tumor subclusters to mesenchymal vSMC cells and endothelial cells as expected (Figure 2C). Similar results were obtained when bulk tumor RNA-seq data were analyzed with single-cell data from benign kidney (Figure S2G). Finally, to bridge the single-cell and snRNA-seq-based predictions, we demonstrated that the PT2 and cluster 29 populations of PT cells published by Lake et al.^35^ were equivalent to the previously identified PT_B and PT_C rare stem-like populations, and we nominated PT2/PT_B cells as the cell-of-origin for several RCC subtypes (Figure S2H). Our analysis supports IC-A as putative cell-of-origin for oncocytomas. Furthermore, we identified the top 100 proteogenomic DE markers in each RCC subtype (Figure S2I; Table S3) and noted their distinct enrichment among benign nephron cell types (Figure 2D), for example MAPRE3 in RO and PIGR in pRCC, which are described in later sections.

### Phosphoproteomic signatures of RCC subtypes and GI tumors

**Figure 3:**
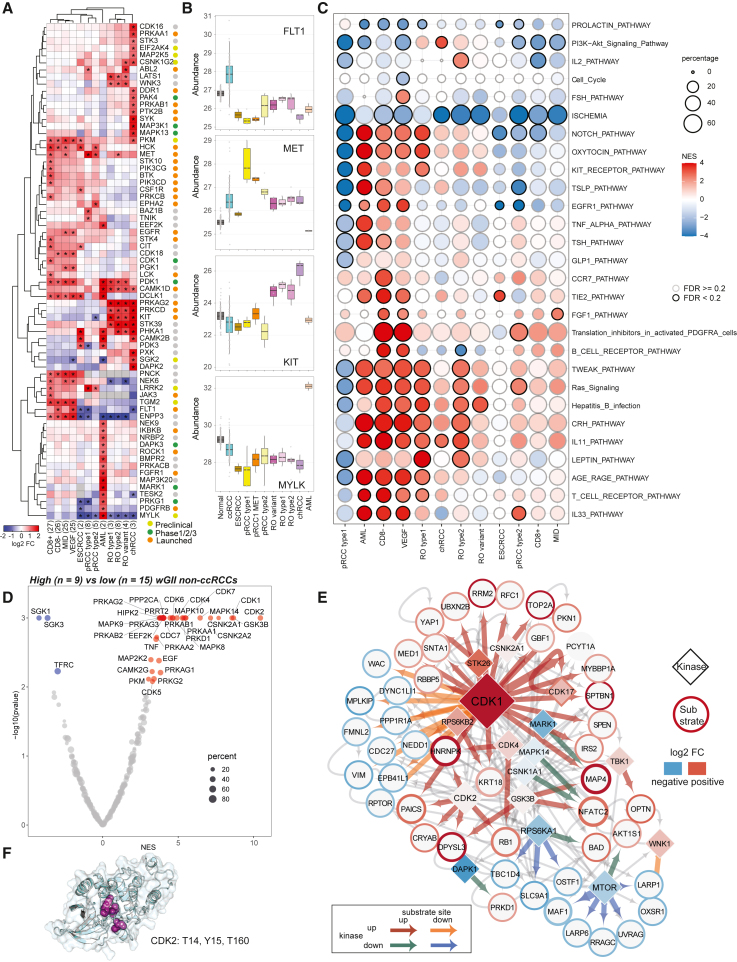
Phosphoproteomic changes in non-ccRCC and genome-unstable tumors (A) DE kinases across major subtypes. Colors represent protein abundance fold change between tumor subtype and NATs. Highlighted kinases are significantly differentially expressed in certain tumor subtypes (adjusted p < 0.01, abs(log2 fc) > 1). CD8+, CD8 positive; CD8–, CD8 negative; MID, metabolic immune-desert; VEGF, VEGF immune-desert. Drug discovery stages (for kinases) from the drug repurposing hub^39^ indicated. (B) Subtype-specific upregulated kinases. Top to bottom: FLT1 in ccRCC, MET in pRCC type 1, KIT in oncocytic tumors, and MYLK in AML. (C) Pathways enriched among the differentially regulated phosphorylation sites across subtypes. Black borders, pathways with FDR < 20%. (D) Kinases that are enriched with down- or upregulated phosphorylation in high compared with low-wGII non-ccRCC. Kinases with enrichment p ≤ 0.05 are labeled. (E) Significantly co-regulated kinase-substrate pairs in high-wGII tumors (FDR < 0.05, abs(log2fc of kinase) > 0.05, abs(log2fc of substrates >0.5)). Diamonds and circles represent kinases and substrate proteins, respectively, and arrows point from former to latter. Diamonds filled with color represent protein abundance log2 fold change between high- and low-wGII non-ccRCC. Border color around circles represents average phosphorylation intensity log2 fold changes between high- and low-wGII non-ccRCC. Size of nodes and thickness of colored arrows are proportional to the number of significant phosphorylation events between kinases and substrate proteins. (F) Protein 3D structure of CDK2. Highlighted residues are significantly upregulated phosphorylation clusters identified by CLUMPS-PTM.

Phosphoproteomics can reveal potentially targetable kinase signaling pathways in tumors. First, as expected we observed enrichment of vascular endothelial growth factor receptor FLT1 in ccRCC,^24^ receptor tyrosine kinases MET and KIT (CD117) in pRCC and chRCC/RO, respectively, and serine threonine kinase MYLK in AML (Figures 3A and 3B; Table S6). In addition, we discovered higher expression of CDK18, NEK6, and PNCK in ccRCC, and BAZ1B and TNIK in pRCC type 1. While LATS1, PRKCD, PRKAG2, and STK39 were common between RO and chRCC, DAPK2, MAPK13, MAP3K1, SYK, DDR1, EIF2AK4, PAK4, and PTK2B were specific to chRCC. Therapeutic inhibition of many of these kinases are currently being evaluated in clinical and preclinical settings (Figure 3A**)**.

Next, to assess phosphorylation changes in subtype-enriched kinases, we compared phospho data and highlighted selected phosphosite changes across the RCC subtypes (Figure S3A; Table S6). For example, phosphorylation of S645 and T507 in the protein kinase C, delta type (PRKCD) is significantly elevated in RO and chRCC compared with pRCCs and ccRCCs (Figure S3A). PRKCD phosphorylation, necessary to prime protein kinase maturation,^40^^,^^41^ is associated with PLC-PKC signaling in leptin stimulation,^42^ which is significantly enriched in RO tumors (Figures 3C and S3A). Leptin regulates the PI3K-AKT pathway, signaling through the JAK-STAT axis^43^ (Figures 3C and S3A). On the other hand, the IL-2 pathway was uniquely upregulated in RO type 2, with high phosphorylation intensity noted in BAD and STAT1 (Figure S3A). Other immune-related pathways including IL-33, TSLP, and T and B cell receptors were generally highly phosphorylated in different immune subtypes of ccRCC and in pRCC, consistent with the snRNA-seq-based observation of higher immune content in ccRCC and pRCC (Figure S2B).

We also explored phosphorylation changes associated with GI, comparing kinase-substrate co-regulation in high- versus low-wGII non-ccRCC samples (STAR Methods; FDR < 0.05, abs(kinase log2 fc) > 0.05, abs(substrate site log2 fc) > 0.5)). Remarkably, cyclin-dependent kinases (CDK1, CDK2) were the most enriched in wGII-high samples (Figures 3D, 3E, and S3D). CDK1 and 2 are critical regulators of multiple steps in cell-cycle and DNA synthesis,^44^ thus closely linked to genomic stability.^45^^,^^46^ Significantly upregulated CDK1 substrates include E2F targets such as RRM2, MCM4, DUT, RFC1, PAICS, NASP, and HMGA1, which regulate DNA replication and chromosome stability^47^ (Figure 3E). Phosphorylation of RB1 T356 by CDK2 (and CDK4/6)^48^^,^^49^ promotes E2F activity^50^ as well as apoptosis in response to replication stress and DNA damage.^51^ Interestingly, CDK2 can also be phosphorylated at Y15 by LYN kinase.^52^ CLUMPS-PTM analysis used to identify phosphorylation clusters in protein 3D structure^53^ revealed three phosphorylation sites in CDK2 (T14, Y15, and T160) forming a phosphorylation hotspot (Figure 3F). Phosphorylation of Y15 and T160 have opposing effects on CDK2 function, Y15 is inhibitory and T160 activating, both events noted together previously in ovarian high-grade serous cancer.^54^ Furthermore, increased phosphorylation of CDK2 Y15 is associated with cell-cycle exit in response to replication stress,^55^^,^^56^ altogether supporting mechanistic links with genomic instability.^46^^,^^56^

### RCC glycoproteome reflects tumor immune infiltration and angiogenesis

**Figure 4:**
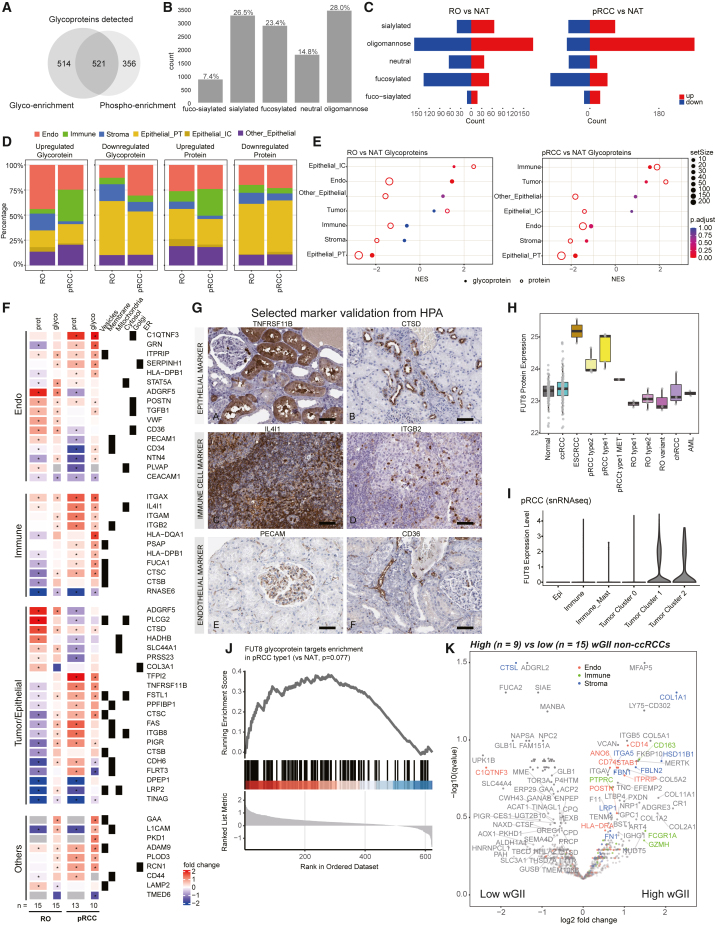
RCC glycoproteome reflects tumor immune infiltration and angiogenesis (A) Glycoprotein overlap between glyco searches on glyco-enriched samples (glyco enrichment) and phospho-enriched samples (phospho enrichment). (B) Distribution of various glycoforms found in the glyco-enriched samples. (C) Distribution of differentially expressed glycoforms. (D) DE glycoproteins (left) and proteins (right) in glyco-enriched samples and their cell type annotation, delineated by cell-type-specific expression from previous scRNA-seq data.^36^ (E) Cell-type enrichment analysis for glycoproteins markers in oncocytoma (left) and pRCC (right) in glyco-enriched samples. (F) DE cell-type-specific glycoprotein markers in glyco-enriched samples. Asterisks indicate significant adjusted q value <0.05) marker expressions. (G) Selected glycoprotein marker expression was validated using data from the Human Protein Atlas. Scale bars, 50 μm. (H) FUT8 protein expression across different RCC subtypes and NATs. (I) FUT8 RNA expression among different cell types identified in type 1 pRCC (C3N-00439) snRNA-seq data. (J) Expression of putative FUT8 glycoprotein targets in pRCC by GSEA. (K) DE glycoproteins (unnormalized data) between high- versus low-wGII non-ccRCC.

Protein glycosylation is linked with cancer development and progression,^57^^,^^58^ as well as tumor microenvironment (TME).^59^ To explore RCC glycobiology and its implications on TMEs, we analyzed two different glycoproteomics^60^ datasets generated independently for this cohort. First, 41 non-ccRCC and 19 NAT samples were enriched for N-glycopeptides,^61^ analyzed by MSFragger-Glyco search pipeline^62^^,^^63^ (STAR Methods). Second, phosphorylation enrichment via immobilized metal affinity chromatography, co-enriched with a substantial number of glycopeptides, particularly sialoglycopeptides,^64^ were analyzed similarly. Our N-linked glycoproteomics pipeline identified 12,503 intact glycopeptides (IGPs) with glycans (glycoforms) from 1,035 glycoproteins in glyco-enriched samples and 29,850 glycoforms from 1,591 glycoproteins in the phospho-enriched samples, respectively, with an overlap of 521 glycoproteins (Figure 4A; Table S7).

Based on glycan monosaccharide composition, IGPs were classified into five categories: oligomannose, sialylated, fucosylated, fuco-sialylated, and neutral moieties.^65^ In glyco-enriched samples, the IGPs were mainly attached to oligomannose glycans, followed by sialylated glycans (Figure 4B), while in phospho-enriched samples IGPs were largely sialylated (Figure S4A) as expected.^64^ Due to sample number constraints, we focused on RO, pRCC, and ccRCC samples. In the glyco-enriched dataset, glycopeptides attached with oligomannose glycans accounted for a large number of the tumor versus normal DE events in both RO and pRCC (Figure 4C). Differential glycosylation abundance positively correlated with corresponding protein abundance changes, but discordant events were also noted (Figures S4B and S4C). Integration with kidney scRNA-seq data^36^ **(**STAR Methods) revealed a significant fraction of the dysregulated glycoproteins contributed by the TME. For example, we observed more upregulated immune compartment changes in pRCC (∼30%) compared with RO (∼5%) (Figures 4D and S4D). Interestingly, only RO samples showed higher fractions of upregulated markers of intercalated cells, a cell type we propose as cell-of-origin for RO. These observations are also consistent with GSEA (Figure 4E). Similar trends of immune infiltration were seen in the phospho-enriched dataset as well for the RO and pRCC samples (Figure S4E). In addition, significant differences between ccRCC immune subtypes were also observed (Figure S4E). As differential glycosylation of key targets has been associated with altered immune and endothelial cell functions,^59^ we looked at selected glycoprotein markers in TME cell types (Figures 4F and S4F). Specifically, RO showed upregulation of IGPs of known marker PLCG2,^66^ as well as ADGRF5 from epithelial/tumor, VWF, POSTN, and STAT5 from endothelial, and CTSD from the immune compartments. On the other hand, pRCC showed upregulation of TFPI2, FSTL1, FAS, and PIGR in the epithelial/tumor, C1QTNF3 and GRN in the endothelial, and ITGAX, HLA-DQA1, IL4I1, and CTSC in the immune compartments. We also observed differential glycosylations not specific to any cell type, for example involving the cancer stem cell marker CD44^67^ in ccRCC and pRCC (Figure 4F). Protein expression of selected markers in different cell types was corroborated by Human Protein Atlas IHC data^68^ (Figure 4G).

Next, evaluating the expression of glycosylation enzymes associated with glycosylation alterations, we noted high levels of glycotransferases (e.g., MGAT1, FUT11) and low levels of glycohydrolases (e.g., GLB1, FUCA1, FUCA2, HEXA, HEXB) in ccRCC versus NATs and other RCC subtypes, at both RNA and protein levels^69^ (Figure S4G). Meanwhile, RO showed upregulated expression of MAN2A1 and ST3GAL1, while pRCC showed higher expression of glycotransferase FUT8^70^^,^^71^ (Figures 4H, 4I, S2B, and S4H). Consistent with FUT8 overexpression, N-glycoproteomics profiling data showed upregulated glycosylation of its putative targets^71^^,^^72^ including CTSC, FSTL1, and LGALS3BP^20^^,^^73^^,^^74^ (Figures 4J and S4I), and MET (Figure S4J), the oncogenic driver of pRCC type 1 classification^75^ and a crucial regulator of EMT.^76^ c-MET (encoded by *MET*) activity is regulated by N-glycosylation.^77^^,^^78^^,^^79^^,^^80^ Upregulation of c-MET glycosylation in pRCC type 1 samples was further localized to MET_N785, recently reported to be largely core-fucosylated^81^ (Figure S4K). Interestingly, L1CAM, which is a FUT8 target and mediator of cancer progression in melanoma,^71^ showed downregulation in this case, suggesting an alternate mechanism in RCCs (Figures 4F and S4F).

Finally, comparing the glycosylation patterns in high- versus low-wGII tumors, immune marker glycoproteins such as GZMA (cytotoxic T cells), FCGR1A, PTPRC (lymphocyte), and CD163 (macrophage), endothelial glycoproteins such as POSTN, ITRIP, ANO6, CD74, CD14, and STAB, stromal markers such as FBN, FBLN2, ITGA5, and COL1A1, and other markers MERTK and FH were enriched in high-wGII tumors (Figure 4K). This supports increased TME cell involvement in high-wGII samples. GZMA is proposed to promote colorectal cancer development.^82^ MERTK is a receptor tyrosine kinase aberrantly expressed in several malignancies and represents a novel target for cancer therapeutics.^83^ GSEA also revealed that glycosylation upregulated in high-wGII samples is involved in EMT hallmark (Table S7), similar to our observation in global proteomics and transcriptomics data (Figure S1H).

### RCC subtypes metabolome delineates tumor growth dynamics

**Figure 5:**
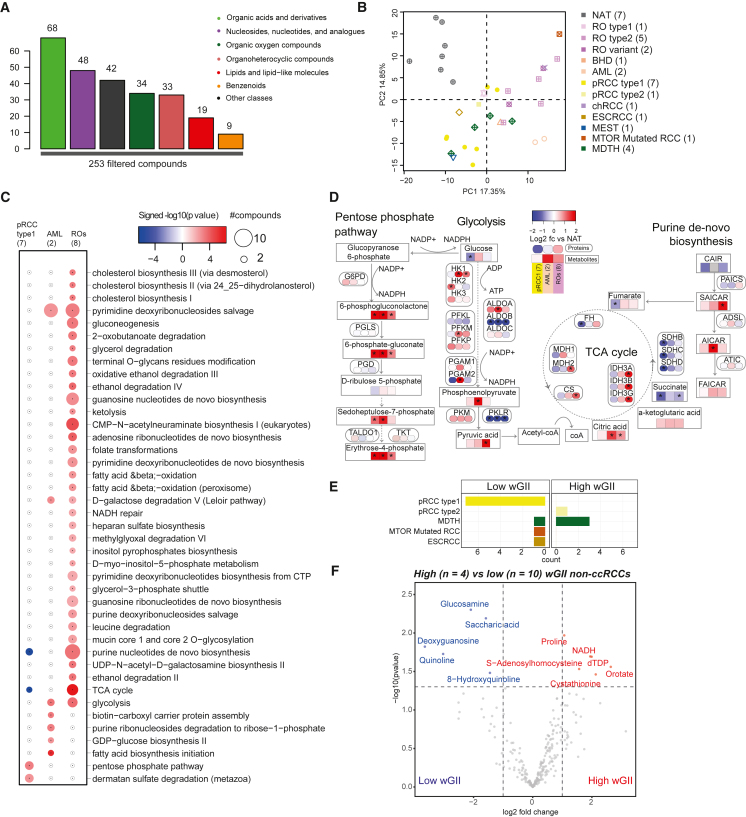
Metabolomic aberrations across RCC subtypes (A) Filtered metabolites analyzed and their distribution across functional categories. (B) Clustering of metabolomics data from different non-ccRCC and NATs. (C) DE pathways between tumor subtypes. Bubble size, number of compounds per pathway. (D) Schematic sketch of key pathways, protein and metabolite abundance log2 fold changes are represented in rounded-corner and regular-corner color boxes, respectively. (E) Distribution of tumor subtypes stratified by high- and low-wGII groups. (F) Metabolites with significant differential abundance (abs(log2fc) > 1 and p < 0.05) between high- and low-wGII tumors.

RCCs are known to exhibit a wide array of mutation-driven metabolic defects.^84^ ccRCCs displaying increased glycolysis and decreased oxidative phosphorylation (Warburg effect) have been associated with high grade, high stage, and low survival.^85^ To explore tumorigenic metabolic reprogramming^86^ in non-ccRCCs, we profiled 253 metabolites across 28 non-ccRCC tumors and 7 NATs (Figure S1A; Table S8). The quantified metabolites include organic acids and derivatives (68), nucleosides, nucleotides, and analogs (48), organic oxygen compounds (42), and other intermediates of major metabolic pathways such as organoheterocyclic compounds, lipids, and benzenoids (Figures 5A and S5A). Differential metabolomic characteristics across RCC subtypes and AML samples were resolved by PCA (Figure 5B), including 65, 136, and 97 differential compounds significantly enriched in pRCC type 1, AML, and ROs, respectively, compared with NATs (≥ 2-fold change and q ≤ 0.05) (Figure S5B). Next, analysis of differentially expressed metabolic enzymes identified metabolic pathways perturbed across RCC subtypes. For example, ccRCC and pRCC type 1 tumors shared some common pathway enrichments compared with ccRCC and ROs (Figure 5C). Specifically, purine nucleotide *de novo* biosynthesis and TCA cycle were depleted in both ccRCC and pRCC type 1 but were enriched in ROs. Pentose phosphate pathway and dermatan sulfate degradation were potentially upregulated in pRCC type 1 but not in other tumor types. Pyrimidine deoxyribonucleoside salvage pathway and glycolysis were active in both AML and ROs. High levels of ACACA, ACACB enzymes, and phosphoric acid in AML indicate increased fatty acid biosynthesis (Figures S5C and S5D).

Several enzymes in the oxidative (e.g., G6PD) as well as non-oxidative (e.g., TALDO1, TKT) phases of pentose phosphate pathway was highly expressed in pRCC type 1 (Figures 5D, S5C, and S5D), associated with increased demand for ribonucleotides in the rapidly proliferating cancer cells.^66^ In renal ROs,^66^ these enzymes are not differentially expressed, likely representing a metabolic barrier to progression. Indeed, ROs showed an accumulation of pyruvate, a product of glycolysis (Figures 5D, S5C, and S5D). Furthermore, low levels of TCA cycle enzymes such as succinate dehydrogenase (SDHB, SDHC, SDHD) and FH are seen in pRCC type 1,^87^ in contrast to high levels of FH, IDH3, and CS seen in ROs. These metabolomic observations are consistent with previously noted high numbers of defective mitochondria (with high abundance of mitochondrial protein) in ROs.^21^^,^^88^^,^^89^

Finally, we compared the metabolomic profile of 4 high-wGII versus 10 low-wGII non-ccRCC samples and identified 6 compounds significantly upregulated and 5 downregulated in the high-wGII group (Figures 5E and 5F**)**. High levels of proline and NADH, coupled with high PYCR1 expression (Figure S1G), indicated higher proline biosynthesis, which might support cancer cell proliferation and survival in oxygen-limiting conditions.^81^ On the other hand, genome-stable samples showed high expression of saccharic acid, glucosamine, and 8-hydroxyquinoline, of which the derivatives are known to have anticancer effects.

### Papillary RCC biomarkers and proteogenomics of activating MET mutations

**Figure 6:**
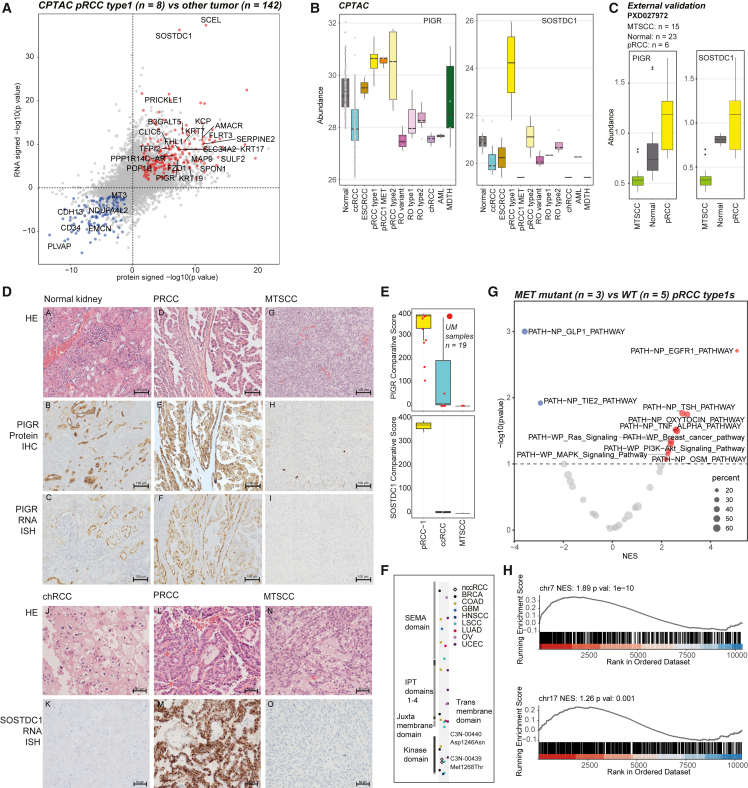
Proteogenomic biomarkers that distinguish pRCC from MTSCC (A) Significantly differential events (abs(log2fc) > 2 and q < 0.05) in protein expression (x axis) and RNA expression (y axis) between pRCC type 1 and other tumors. (B) Specificity of pRCC type 1 protein markers PIGR and SOSTDC1. (C) Expression of pRCC type 1 protein markers PIGR and SOSTDC1 in the proteomics data from Xu et al.^23^ (PXD027972). (D) H&E, protein IHC, and RNA-ISH images (top to bottom) of biomarker PIGR in normal kidney tissue, pRCC, MTSCC tumors (upper panels from left to right) and SOSTDC1 in chRCC, pRCC, and MTSCC (lower panels from left to right). (E) RNA-ISH comparative scores of PIGR and SOSTDC1 in different tumor types. Red points represent external University of Michigan samples. (F) Location of missense mutations in MET across TCGA cohorts are colored on the MET protein domain diagram. (G) PTM-SEA analysis shows pathways such as EGFR are significantly enriched with increased phosphorylation in MET mutant pRCC samples. (H) Enrichment in chromosomes 7 and 17 gene sets are tested with protein expression difference between chromosome 7 gain and no gain non-ccRCC sample groups.

Malignant pRCCs^75^ accounting for 15% of all RCCs are histomorphologically and genetically heterogeneous tumors that currently lack specific diagnostic biomarkers. Importantly, a subset of pRCCs show overlapping morphology with mucinous tubular and spindle cell carcinoma (MTSCC), a rare benign tumor^90^ confounding clinical care decisions. To delineate pRCC-specific biomarkers using our multi-omics data, we identified a number of pRCC type 1-specific candidates significantly upregulated (n = 176, log2 fc > 1, q < 0.05) and downregulated (n = 108, log2 fc < −1, q < 0.05) (Figure 6A; Table S9). Top pRCC-specific candidates included sclerostin domain-containing protein1 (SOSTDC1)^91^ and polymeric immunoglobulin receptor (PIGR), further validated in the pan-RCC RNA-seq data from a combined cohort of TCGA plus MCTP (n = 1,000) (Figures 6B, S6A, and S6B). Comparing MTSCC (n = 18) and pRCC (n = 8) proteomics data from Xu et al.^23^ we noted both PIGR and SOSTDC1 proteins highly upregulated in pRCC compared with MTSCC (Figure 6C), and we validated these findings by IHC and RNA-ISH (Figures 6D and 6E). SOSTDC1 as a biomarker for pRCC has not been studied previously. PIGR has been listed as pRCC-specific in Jorge et al.’s DE analysis,^20^ corroborated by our observations.

Next we explored the proteogenomic impact of activating mutations in the MET kinase domain frequently observed in type 1 pRCC. Two type 1 pRCC samples with hotspot mutations in the *MET* kinase domain (Asp1246Asn and Met1268Thr) (Figure 6F), compared against type 1 pRCC cases with wild-type MET (n = 5), revealed upregulated phosphoserine/threonine and phosphotyrosine events, including several known MET substrates such as GAB1 Y689 (Figure S6C). In addition to known MET substrates, enrichment analysis with PTM-SEA identified signaling pathways enriched with upregulated phosphorylation sites, such as EGFR, PI3K-AKT, and MAPK (Figure 6G). The intracellular signaling cascades activated by MET include the PI3K-AKT, RAC1-cell division control protein CDC42, RAP1, and RAS-MAPK pathways. Cooperative signaling between MET and EGFR has been observed during kidney development,^92^ and aberrant cross-signalling in renal cancer noted to have major implications for therapy.^93^

chr7 gain is common in type 1 pRCC and occurs in some ccRCCs. Increased abundance of chr7 genes is notably observed on RNA level (Figures S6D and S6E). As both chr7/17 gains tend to co-occur in pRCC, we also saw increased chr17 gene expression in non-ccRCC tumors, which was not observed in ccRCC (Figures 6H and S6F). Pathway enrichment analysis revealed upregulation in EMT, angiogenesis and KRAS signaling, and downregulation in adipogenesis and fatty acid metabolism in both ccRCC and non-ccRCC tumors with chr7 gain (Figure S6G). In addition, papillary lineage tumors exhibiting chr7 gain show elevated phosphorylation activities associated with several chr7 kinases, including HIPK2, CDK13, MET, CDK6, and BRAF (Figure S6H).

### Regulons and differential diagnosis biomarkers in oncocytic tumors

Current IHC clinical markers for chRCC^17^^,^^94^ and RO^95^^,^^96^ include KRT7, CD117 (c-kit), epithelial mesenchymal antigen, parvalbumin, S100A, and kidney-specific cadherins. Instances of patchy staining and pattern overlap with chRCC^17^^,^^94^^,^^97^ are limitations, as in clinical diagnostic criteria, patchy KRT7 expression usually supports RO, while strong uniform staining is usually supportive of chRCC.

**Figure 7:**
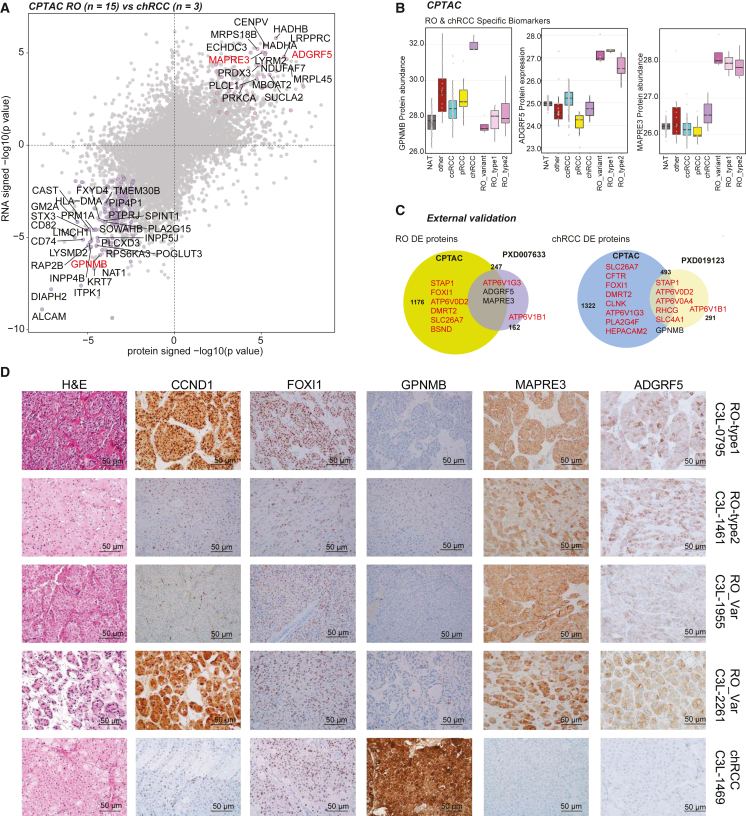
Proteogenomic biomarkers that distinguish oncocytomas (RO) from chRCC (A) DE proteins (x axis) and mRNA (y axis) between RO and chRCC. Indicated genes have p < 0.01 in both dimensions, and candidates in red (MAPRE3, ADGRF5, GPNMB) were subsequently validated as RO- and chRCC-specific biomarkers, respectively. (B) chRCC marker GPNMB (left) and RO biomarkers ADGRF5 and MAPRE3 protein abundance in different subtypes. (C) Overlap between DE proteins identified in this study (CPTAC) and the publicly available PXD007633 dataset in RO (left) and PXD019123 chRCC dataset (right). Genes in red are associated with FOX1 and DMRT2. (D) Immunohistochemistry validation of nominated markers seen in representative tumor sections. Corresponding H&E staining images are shown alongside.

KRT7 was higher in chRCC (both RNA and protein levels), markers such as KIT and FOXI1 were equally expressed in ROs and chRCC, while CCND1 overexpression was specific to fusion-positive RO (Figure S7A). We next employed SCENIC tool,^98^^,^^99^ which examines transcriptional modules or regulons (coexpression of a given transcription factor and its target genes) to characterize differences among the different tumor and benign tissues (Figure S7B). The transcription factor FOXI1 is specifically expressed in intercalated cells and tumors such as chRCC and ROs.^8^^,^^100^ Regulons shared between chRCC and RO include the lineage-specific transcription factors FOXI1 and DMRT2, and DMRT2 is a known target of FOXI1. We also identified several regulons that were enriched only in chRCC, such as ZBTB7A, SMARCB1, E4F1, and FOXJ2, among others (Figure S7B) that were not previously associated with this disease. We next performed RNA and protein DE analysis between three chRCC and 15 ROs profiled in this study to identify diagnostic biomarkers (Figure 7A). Compared with two publicly available datasets, the CPTAC proteomic dataset had a better coverage of FOXI1 and DMRT2 where both transcription factor proteins and most of their gene targets (such as ATPV0D2, HEPACAM2, DMRT2, etc.) showed differential expression in tumor versus normal comparisons, as expected (Figure 7A; Table S10).^66^^,^^96^

DE analysis (Figure 7A) discovered candidates such as microtubule-associated protein RP/EB family member 3, adhesion G protein-coupled receptor F5 (MAPRE3, ADGRF5, specific to RO) and glycoprotein nonmetastatic melanoma protein B (GPNMB upregulated in chRCC) (Figures 7B, 7C, and S7C). We validated our findings in independent publicly available mass spectrometry-based proteomics data for RO (n = 6, PXD007633) and chRCC (n = 9, PXD019123) (Figure 7C). Using IHC, we next independently confirmed and validated biomarker specificity including CCND1 protein overexpression in gene fusion-positive ROs, MAPRE3, and ADGRF5 (also identified in the glycoproteomics analysis) expression in all RO subtypes (Figure 7D) and GPNMB in chRCC. While CCND1 and FOXI1 were enriched in the nuclei, GPNMB showed a homogeneous and moderate/strong expression within the cytoplasmic compartment of the chRCC tumor cells, and MAPRE3 protein showed a predominant membranous expression pattern in RO. ADGRF5, also called GPR116, is an adhesion G protein-coupled receptor, and an emerging role in cancers for this family of proteins is being investigated.^101^ Furthermore, we observed THSD4 upregulation in RO type 1, but downregulation in RO type 2 compared with NAT (Figure S7D), future validation of this marker might enable rapid distinction between the two subtypes.

## Discussion

NGS and global proteomics data generated by CPTAC provide a high-quality data resource that can be explored further to derive novel biomarkers and gain deeper insights into disease biology. Motivated by the specific clinical need of biomarkers specific to rare subtypes of renal cell carcinoma, we carried out multi-omics analyses to identify protein/mRNA biomarkers to distinguish benign ROs from chRCC (MAPRE3, ADGRF5, GPNMB), pRCC from MTSCC (SOSTDC1, PIGR), and tumors with high wGII (PYCR1, IGF2BP3). A number of these markers were validated by IHC and RNA-ISH, supporting further evaluation in independent cohorts to facilitate development of renal cancer biomarker panels for clinical use.

A number of single-cell studies in renal cancers have mainly characterized ccRCC focusing on immune infiltration,^102^^,^^103^^,^^104^ immunotherapy resistance,^105^ and cell-of-origin,^36^^,^^106^ while non-ccRCC tumors largely remain uncharacterized. Here, we analyzed snRNA-seq data from eight non-ccRCC samples covering all oncocytoma subtypes. Our analysis highlights intra-tumor transcriptomic heterogeneity and a wide variation in the degree of immune infiltration among non-ccRCC subtypes, wherein malignancies such as pRCC and AML showed higher levels of immune infiltration compared with chRCC, ROs, TRCC, and others (Figure S2A). We also identified several cell-type-specific markers representing putative cell-of-origin that could be further characterized for expansion of diagnostic panels.

Some non-ccRCC tumors have been previously profiled proteomically,^23^^,^^66^^,^^96^ but the landscape of their PTMs remains uncharacterized. Here, we examined two different PTM profiles, namely protein phosphorylations and glycosylations from non-ccRCC and ccRCC samples. Besides identifying known kinase expression patterns and therapeutic targets in RCC subtypes such as FLT1 (ccRCC), KIT (chRCC and RO), and MET (pRCC type 1), we have identified several additional subtype-enriched kinases, with some among them being evaluated for their therapeutic utility in preclinical and clinical settings. Additional characterization studies of these potential kinase targets to evaluate their therapeutic utility is warranted. Our integrative phosphoproteomics analysis on tumors with GI besides identifying important biomarkers for early detection of this molecular subset, clarifies signaling cascades that might drive this molecular disease subset. We show significantly increased cyclin-dependent kinase activities in GI tumors, which suggests increased proliferation (taken together with pathways found enriched at whole-protein and RNA abundance) and decreased MTOR activity in these tumors. The latter observation now provides a reason, at least in part, on why MTOR inhibitors such as everolimus show poor response in metastatic RCC.

To explore RCC glycobiology and its implication on TMEs, we analyzed both phospho-glyco (contained more sialylated events) and glyco-enriched (had more oligomannose IGPs) data. Using cell type gene expression annotation from previous single-cell data, we inferred TME contribution within the differentially expressed glycoproteins*.* Larger impact of TME was noted in both ccRCC and pRCC compared with ROs, revalidating the biology of these tumors. Further core-fucosylation characterization using the glyco data generated here, might deepen our understanding of tumor and immune microenvironment in pRCC. Finally, differential glycosylation events noted on proteins potentially contributed by the TME compartment in higher wGII non-ccRCC support higher immune infiltration.

Genomic drivers of RCC are linked to dysregulated metabolism.^107^ Interesting similarities and differences observed among kidney tumor subtypes include the depletion of purine nucleotide *de novo* biosynthesis and TCA cycle intermediates in ccRCC and pRCC type 1 tumors, and, in contrast, their enrichment in ROs. Enrichment of the pentose phosphate pathway and dermatan sulfate degradation in pRCC type 1, oncometabolite SAICAR in ROs, and proline and NADH, coupled with high PYCR1 expression in non-ccRCC tumors with high wGII warrant further investigations.

In conclusion, proteogenomic analysis provided insights into a variety of non-ccRCC subtypes, identifying histologically specific diagnostic biomarkers, markers of GI, and revealed the interconnectedness between the omic layers. While single-nucleus analysis highlighted the potential intra-tumor heterogeneity and differences in putative cell-of-origin within non-ccRCC subtypes. Fundamentally, this study provides a comprehensive proteogenomic data resource to enable further in-depth exploration of the biology of these rare kidney tumors.

### Limitations of the study

This study evaluates a wide range of non-ccRCC subtypes with an extensive array of multi-omic analyses but has its limitations. The specific tissue procurement protocols necessary to facilitate high-quality protein-based multi-omics limited this study to prospective sample recruitment, thereby limiting the number of tumors analyzed. Lack of samples representing other non-ccRCC subtypes such as FH-deficient RCC, clear cell pRCC, among others, due to nonavailability of those rare subtypes is another limitation. Some subtypes are represented by one or two samples, and do not account for any heterogeneity within these subtypes. However, currently there is little or no high-quality multi-omics data available for most of these tumor subtypes, therefore observations presented here can represent a foundation for further, targeted analyses of specific features. This future work will be essential for confirming and refining this study’s observations, which serve as an initial stepping stone for a deeper understanding of the complexity of non-ccRCCs.

## Consortia

The members of the National Cancer Institute Clinical Proteomic Tumor Analysis Consortium are Eunkyung An, Shankara Anand, Andrzej Antczak, Alexander J. Lazar, Meenakshi Anurag, Jasmin Bavarva, Chet Birger, Michael J. Birrer, Melissa Borucki, Shuang Cai, Anna Calinawan, Wagma Caravan, Steven A. Carr, Daniel W. Chan, Feng Chen, Lijun Chen, Siqi Chen, David Chesla, Arul M. Chinnaiyan, Hanbyul Cho, Seema Chugh, Marcin Cieslik, Sandra Cottingham, Reese Crispen, Felipe da Veiga Leprevost, Aniket Dagar, Saravana M. Dhanasekaran, Rajiv Dhir, Li Ding, Marcin J. Domagalski, Brian J. Druker, Nathan J. Edwards, David Fenyö, Stacey Gabriel, Gad Getz, Yifat Geffen, Michael A. Gillette, Charles A. Goldthwaite Jr., Anthony Green, Shenghao Guo, Jason Hafron, Sarah Haynes, Tara Hiltke, Barbara Hindenach, Bart Williams, Katherine A. Hoadley, Alex Hopkins, Noshad Hosseini, Galen Hostetter, Andrew Houston, Yi Hsiao, Scott D. Jewell, Xiaojun Jing, Ivy John, Corbin D. Jones, Karen A. Ketchum, Iga Kołodziejczak, Chandan Kumar-Sinha, Anne Le, Toan Le, Ginny Xiaohe Li, Yize Li, W. Marston Linehan, Tao Liu, Yin Lu, Jie Luo, Weiping Ma, Avi Ma’ayan, D.R. Mani, Rahul Mannan, Peter B. McGarvey, Rohit Mehra, Mehdi Mesri, Nataly Naser Al Deen, Alexey I. Nesvizhskii, Chelsea J. Newton, Kristen Nyce, Gilbert S. Omenn, Amanda G. Paulovich, Samuel H. Payne, Francesca Petralia, Daniel A. Polasky, Sean Ponce, Barb Pruetz, Ratna R.Thangudu, Boris Reva, Christopher J. Ricketts, Ana I. Robles, Karin D. Rodland, Henry Rodriguez, Eric E. Schadt, Michael Schnaubelt, Yvonne Shutack, Richard D. Smith, Mathangi Thiagarajan, Pamela VanderKolk, Negin Vatanian, Josh Vo, Pei Wang, Xiaoming Wang, George Wilson, Maciej Wiznerowicz, Fengchao Yu, Kakhaber Zaalishvili, Cissy Zhang, Hui Zhang, Yuping Zhang, Stephanie Miner, Bing Zhang, Zhen Zhang, and Xu Zhang.

## STAR★Methods

### Key resources table

**Table:** 

REAGENT or RESOURCE	SOURCE	IDENTIFIER
**Antibodies**		

Goat Polyclonal IgG Human Osteoactivin/GPNMB antibody	R&D Systems	Catalog: AF2550, RRID: AB_416615
Rabbit Polyclonal IgG Human MAPRE3 antibody	Atlas Antibodies	Catalog: HPA009263 RRID: AB_1078716
Mouse Monoclonal IgG Human FOXI1 antibody	Origene Technologies	Catalog: TA800146 RRID: AB_2625262
Rabbit Monoclonal IgG Human Cyclin D1	Cell Marque	Catalog: 241R-18 RRID: AB_1158233
Mouse Monoclonal IgG Human PIGR antibody	Santa Cruz	Catalog: SC-374343, RRID: AB_10989564
Rabbit Polyclonal IgG Human PYCR1 antibody	Cell Signaling Technology	Catalog: 47935
Rabbit Polyclonal IgG Human AKT antibody	Cell Signaling Technology	Catalog: 9272
Rabbit Polyclonal IgG Human Phospho-AKT (Ser473) Antibody	Cell Signaling Technology	Catalog: 9271
Rabbit Polyclonal IgG Human p44/42 MAPK (Erk1/2) Antibody	Cell Signaling Technology	Catalog: 9102
Rabbit Monoclonal IgG Human Phospho-p44/42 MAPK (Erk1/2) (Thr202/Tyr204) antibody	Cell Signaling Technology	Catalog: 4376
Rabbit Polyclonal IgG Human Vinculin Antibody	Cell Signaling Technology	Catalog: 4650

**Biological samples**		

Primary tumor and normal adjacent tissue samples	See experimental model and study participant details	See Table S1

**Critical commercial assays**		

Discovery CC1	Roche-Ventana Medical System	Catalog: 950-500
Discovery CC2	Roche-Ventana Medical System	Catalog: 950-123
OptiView Universal DAB Detection Kit	Roche-Ventana Medical System	Catalog: 760-700
UltraView Universal DAB Detection Kit	Roche-Ventana Medical System	Catalog: 760-500
Discovery mRNA DAB Detection RUO	Roche-Ventana Medical System	Catalog: 760-224
RNAscope® 2.5 HD Reagent Kit -BROWN	Advanced Cell Diagnostics, Inc	Catalog: 322300
RNAscope® VS Universal HRP Reagent Kit	Advanced Cell Diagnostics, Inc	Catalog: 323200
RNAscope Target Probe - Hs-PIGR	Advanced Cell Diagnostics, Inc	Catalog: 472681
RNAscope Target Probe - Hs-PYCR1	Advanced Cell Diagnostics, Inc	Catalog: 509259
RNAscope Target Probe - Hs-SOSTDC1	Advanced Cell Diagnostics, Inc	Catalog: 469929
RNAscope PositiveProbe - Hs-PPIB	Advanced Cell Diagnostics, Inc	Catalog: 313901/313909
RNAscope Negative Probe – DapB	Advanced Cell Diagnostics, Inc	Catalog: 310043/312039
Rabbit Polyclonal IgG Human IGF2BP3	Proteintech	Catalog: 14642-1-AP, RRID: AB_2122782
Rabbit Polyclonal IgG Human ADGRF5 (GPR116)	Proteintech	Catalog: 14047-1-AP, RRID: AB_2113095

**Deposited data**		

CPTAC non-ccRCC clinical data and proteomic data	This manuscript	https://pdc.cancer.gov/
CPTAC ccRCC genomic, transcriptomic, and snRNA-seq data	This manuscript	https://portal.gdc.cancer.gov/projects/CPTAC-3
CPTAC non-ccRCC pathology and radiology images	This manuscript	https://portal.imaging.datacommons.cancer.gov/
TCGA KIRC	Cancer Genome Atlas Research Network^108^	https://portal.gdc.cancer.gov/
TCGA KIRP	Cancer Genome Atlas Research Network^75^	https://portal.gdc.cancer.gov/

**Software and algorithms**		

CNVEX		https://github.com/mctp/cnvex
R v4.1	R Development Core Team	https://www.R-project.org
Python	Python Software Foundation	https://www.python.org/
Philosopher	da Veiga Leprevost et al.^109^	https://philosopher.nesvilab.org/
MSFragger	Kong et al.^110^	https://msfragger.nesvilab.org/
PTM-Shepherd	Geiszler et al.^111^	https://ptmshepherd.nesvilab.org/
TMT-Integrator	Djomehri et al.^112^	https://github.com/Nesvilab/TMT-Integrator
ARD-NMF	Tan et al.^30^	https://github.com/getzlab/getzlab-SignatureAnalyzer
CancerSubtypes	Xu et al.^113^	https://www.bioconductor.org/packages/release/bioc/html/CancerSubtypes.html
Limma	Ritchie et al.^90^	https://bioconductor.org/packages/release/bioc/html/limma.html
PTM-SEA	Krug et al.^114^	https://github.com/broadinstitute/ssGSEA2.0
KSA-2D	Han et al.^115^	https://github.com/ginnyintifa/KSA2D
CLUMPS-PTM	Geffen et al.^53^	https://github.com/getzlab/CLUMPS-PTM
IMPaLA	Kamburov et al.^116^	http://impala.molgen.mpg.de/
ClusterProfiler	Yu et al.^117^	https://bioconductor.org/packages/release/bioc/html/clusterProfiler.html
pySCENIC	Van de Sande et al.^118^	https://github.com/aertslab/pySCENIC
BayesDeBulk	Petralia et al.^119^	http://www.bayesdebulk.com/
DreamAI	Ma et al.^120^	https://github.com/WangLab-MSSM/DreamAI
OmniPathR	D Turei et al.^99^^,^^121^	https://www.bioconductor.org/packages/release/bioc/html/OmnipathR.html
Survival	Therneau et al.^122^	https://cran.r-project.org/web/packages/survival/index.html

### Resource availability

#### Lead contact

Further information and requests for resources and reagents should be directed to and will be fulfilled by the Lead Contact, Alexey Nesvizhskii, nesvi@med.umich.edu.

#### Materials availability

This study did not generate new unique reagents.

#### Data and code availability

• Clinical data and raw proteomic data reported in this paper can be accessed via the CPTAC Data Portal at: https://cptac-data-portal.georgetown.edu/cptac. Genomic, transcriptomic, and snRNA seq data files can be accessed via Genomic Data Commons (GDC) at: https://portal.gdc.cancer.gov/projects/CPTAC-3. Proteomic data files can be accessed via Proteomic Data Commons (PDC) at: https://pdc.cancer.gov/with following accession codes: PDC000464, PDC000465, and PDC000466. Processed data used in this publication can be found in the CPTAC PDC. An interactive ProTrack web portal^30^ is also provided to visualize multi-omics data in interactive heatmap and boxplot visualizations, as well reviewing histological images, exploring the cohort with a sample dashboard, and reviewing quality control results for ccRCC and non-ccRCC data (http://ccrcc-conf.cptac-data-view.org/).
• This paper does not report original code.
• Any additional information required to reanalyze the data reported in this work paper is available from the STAR Methods upon request.

### Experimental model and study participant details

#### Human subjects

A total of 151 participants were included in this study.Institutional review boards at each Tissue Source Site (TSS) reviewed protocols and consent documentation, in adherence to Clinical Proteomic Tumor Analysis Consortium (CPTAC) guidelines.

#### Clinical data annotation

Clinical data were obtained from TSS and aggregated by the CPTAC Biospecimen Core Resource (BCR, at the Pathology and Biorepository Core of Van Andel Research Institute (Grand Rapids, MI)). Data forms were stored as Microsoft Excel files (.xls). Clinical data can be accessed and downloaded from the CPTAC Data Portal. Demographics of patients can be viewd at ProTrack (http://ccrcc-conf.cptac-data-view.org/). Patients with any prior history of other malignancies within twelve months or any systemic treatment (chemotherapy, radiotherapy, or immune-related therapy) were excluded from this study.

### Method details

#### Sample processing

The CPTAC BCR manufactured and distributed biospecimen kits to the TSS located in the US, and Europe. Each kit contains a set of pre-manufactured labels for unique tracking of every specimen respective to TSS location, disease, and sample type, used to track the specimens through the BCR to the CPTAC proteomic and genomic characterization centers. Tissue specimens averaging 200 mg were snap-frozen by the TSS within a 30 min cold ischemic time (CIT) (CIT average = 15 min) and an adjacent segment was formalin-fixed paraffin embedded (FFPE) and H&E stained by the TSS for quality assessment to meet the CPTA tissue requirements. Routinely, several tissue segments for each case were collected. Tissues were flash-frozen in liquid nitrogen (LN2) and then transferred to a liquid nitrogen freezer for storage until approval for shipment to the BCR. Specimens were shipped using a cryoport that maintained an average temperature of under −140°C to the BCR with a time and temperature tracker to monitor the shipment. Receipt of specimens at the BCR included a physical inspection and review of the time and temperature tracker data for specimen integrity, followed by barcode entry into a biospecimen tracking database.

Specimens were again placed in LN2 storage until further processing. Acceptable non-ccRCC tumor tissue segments were determined by TSS pathologists based on the percent viable tumor nuclei (>80%), total cellularity (>50%), and necrosis (<20%). Segments received at the BCR were verified by BCR and Leidos Biomedical Research (LBR) pathologists and the percent of the total area of tumor in the segment was also documented. Additionally, disease-specific working group pathology experts reviewed the morphology to clarify or standardize specific disease classifications and correlation to the proteomic and genomic data. The cryopulverized specimen was divided into aliquots for DNA (30 mg) and RNA (30 mg) isolation and proteomics (50 mg) for molecular characterization. Nucleic acids were isolated and stored at −80°C until further processing and distribution; cryopulverized protein material was returned to the LN2 freezer until distribution. Shipment of the cryopulverized segments used cryoports for distribution to the proteomic characterization centers and shipment of the nucleic acids used dry ice shippers for distribution to the genomic characterization centers; a shipment manifest accompanied all distributions for the receipt and integrity inspection of the specimens at the destination.

#### Sample cohort details

In this study, we performed proteogenomics profiling of 194 tumor and NAT samples from the discovery cohort^27^ (110 tumors profiled with proteomics and RNA-seq, 84 NATs profiled with proteomics and 73 NATs profiled with RNA-seq), 4 samples from confirmatory^13^ (2 tumors and 2 NATs profiled with both proteomics and RNA-seq) and 56 samples from non-ccRCC cohorts (39 tumors profiled with proteins and RNA-seq, 17 NATs profiled with proteomics and 14 NATs profiled with RNA-seq). Within the 110 tumor samples from the discovery ccRCC cohort,^27^ 103 were confirmed ccRCC and 7 were non-ccRCC.

Across all three cohorts, we profiled 103 ccRCC tumor samples (all from the discovery cohort) and 48 non-ccRCC tumor samples (7 samples from the discovery cohort, 2 samples from the confirmatory cohort, 39 from the non-ccRCC cohort). Within the 48 non-ccRCC samples, we counted 15 ROs (3 RO type 1, 8 RO type 2, 4 RO variant), 13 papillary RCC (pRCC, 8 pRCC with the previous defined type 1 features (pRCC-1) and 5 without (pRCC-2)), 3 chromophobe RCC (chRCC), 2 angiomyolipoma (AML), 2 eosinophilic solid and cystic RCC (ESCRCC), 1 Birt-Hogg-Dube syndrome-associated renal cell carcinoma (BHD), 1 mixed epithelial and stromal tumor of the kidney (MEST), 1 MTOR mutated RCC, 1 translocation RCC (TRCC), 8 molecularly divergent to histology RCC (MDTH), and 1 plasmacytoid urothelial carcinoma (PUC). The following three samples were excluded from all downstream analysis: 2 NAT samples (C3*N*-00314-N and C3*N*-01524-N) that were found to be contaminated with tumor tissue and 1 PUC sample (C3L-02212-T) which is not a renal cell carcinoma. One tumor (C3*N*-02204-T) indicated with asterix in Figure 1A is excluded from the wGIIanalysis since genomics data was not fully available at the time of data freeze.

#### Immunohistochemistry (IHC)

Immunohistochemistry (IHC) was performed on 4-micron formalin-fixed, paraffin-embedded (FFPE) tissue sections. The Ventana Benchmark XT staining platform with Discovery CCI and CC2 (Ventana cat#950-500 and 950-123) were used for antigen retrieval. The immune complexes were developed with either the ultraView or optiView Universal DAB (diaminobenzidine tetrahydrochloride) Detection Kit (Ventana cat#760-500 and cat#760-700). he details of the panel of primary antibodies utilized is as follows: polymeric immunoglobulin receptor (PIGR/Anti-SC; Santa Cruz, mouse monoclonal, catalog no. SC-374343), cyclin D1 (CCND1; Cell Marque, rabbit monoclonal, catalog no. 241R-18), transmembrane glycoprotein NMB (GPNMB, R&D systems, goat polyclonal, catalog no. AF2550), microtubule-associated protein RP/EB family member-3 (MAPRE3, Atlas antibodies, rabbit polyclonal, catalog no. HPA009263), and forkhead boxI1 (FOXI1, Origene antibodies, mouse monoclonal, catalog no. TA800146). Brown pigmentation within the subcellular component (cytoplasmic and or membranous for PIGR, GPNMB, MAPRE3 and nuclear for FOXI1 and CCND1) were taken as positive expressions. In addition for PIGR the presence and intensity of cytoplasmic staining were scored where the percentage of PIGR positive neoplastic cells and the staining intensity (none, 0; weak, 1; moderate, 2; strong, 3) were recorded for each tumor as described previously.^8^ Appropriate positive and negative control tissue were run in each assay batch.

#### RNA *in situ* hybridization (RNA-ISH)

RNA-ISH was performed using the RNAscope 2.5 HD Brown kit (Advanced Cell Diagnostics, Newark, CA) and target probes against PIGR (472681 Hs-PIGR targeting NM_002644.3, 2-903nt), PYCR1 (509259 Hs-PYCR1 targeting NM_001282281.1, 64-1770nt), and SOSTDC1 469929 Hs-SOSTDC1 targeting NM_015464.2, 2-938nt) according to the manufacturer’s instructions. RNA quality was evaluated in each case utilizing a positive and a negative control probe against human housekeeping gene Peptidylprolyl Isomerase B (PPIB) (313901 for manual and 313909 for Ventana automated system) and bacillus bacterial gene DapB (310043 for manual and 312039 for Ventana automated system) respectively. The assay was run

according to the protocol previously described.^6^^,^^9^

Stained slides were evaluated under a light microscope at ×100 and ×200 magnification for RNA-ISH signals in neoplastic cells by multiple study investigators. Each RNA molecule in this assay’s result is represented as a punctate brown dot. The expression level was evaluated according to the RNAscope scoring criteria: score 0 = no staining or <1 dot per 10 cells; score 1 = 1–3 dots per cell, score 2 = 4–9 dots per cell, and no or very few dot clusters; score 3 = 10–15 dots per cell and <10% dots in clusters; score 4 = >15 dots per cell and >10% dots in clusters. The H-score was calculated for each examined tissue section as the sum of the percentage of cells with score 0–4 [(A% × 0) + (B% × 1) + (C% × 2) + (D % × 3) + (E% × 4), A + B + C + D + E = 100], using previously published scoring criteria.^6^^,^^9^

#### Sample processing for genomic DNA and total RNA extraction

Our study sampled a single site of the primary tumor from surgical resections, due to the internal requirement to process a minimum of 125 mg of tumor issue and 50 mg of adjacent normal tissue. DNA and RNA were extracted from tumor and blood normal specimens in a co-isolation protocol using Qiagen’s QIAsymphony DNA Mini Kit and QIAsymphony RNA Kit. Genomic DNA was also isolated from peripheral blood (3–5 mL) to serve as matched normal reference material. The Qubit dsDNA BR Assay Kit was used with the Qubit 2.0 Fluorometer to determine the concentration of dsDNA in an aqueous solution. Any sample that passed quality control and produced enough DNA yield to go through various genomic assays was sent for genomic characterization. RNA quality was quantified using both the NanoDrop 8000 and quality assessed using Agilent Bioanalyzer. A sample that passed RNA quality control and had a minimum RIN (RNA integrity number) score of 7 was subjected to RNA sequencing. Identity match for germline, normal adjacent tissue, and tumor tissue was assayed at the BCR using the Illumina Infinium QC array. This beadchip contains 15,949 markers designed to prioritize sample tracking, quality control, and stratification.

#### Preparation of libraries for cluster amplification and WGS sequencing

An aliquot of genomic DNA (350 ng in 50 μL) was used as the input into DNA fragmentation (aka shearing). Shearing was performed acoustically using a Covaris focused-ultrasonicator, targeting 385bp fragments. Following fragmentation, additional size selection was performed using an SPRI cleanup. Library preparation was performed using a commercially available kit provided by KAPA Biosystems (KAPA Hyper Prep without amplification module) and with palindromic forked adapters with unique 8-base index sequences embedded within the adapter (purchased from IDT). Following sample preparation, libraries were quantified using quantitative PCR (kit purchased from KAPA Biosystems), with probes specific to the ends of the adapters. This assay was automated using Agilent’s Bravo liquid handling platform. Based on qPCR quantification, libraries were normalized to 1.7 nM and pooled into 24-plexes.

#### Cluster amplification and sequencing (HiSeq X)

Sample pools were combined with HiSeq X Cluster Amp Reagents EPX1, EPX2, and EPX3 into single wells on a strip tube using the Hamilton Starlet Liquid Handling system. Cluster amplification of the templates was performed according to the manufacturer’s protocol (Illumina) with the Illumina cBot. Flow cells were sequenced to a minimum of 15x on HiSeq X utilizing sequencing-by-synthesis kits to produce 151bp paired-end reads. Output from Illumina software was processed by the Picard data processing pipeline to yield BAMs containing demultiplexed, aggregated, aligned reads. All sample information tracking was performed by automated LIMS messaging.

#### Whole exome sequencing library construction

Library construction was performed as described in Fisher et al.,^123^ with the following modifications: initial genomic DNA input into shearing was reduced from 3 μg to 20–250 ng in 50 μL of solution. For adapter ligation, Illumina paired-end adapters were replaced with palindromic forked adapters, purchased from Integrated DNA Technologies, with unique dual-indexed molecular barcode sequences to facilitate downstream pooling. Kapa HyperPrep reagents in 96- reaction kit format was used for end repair/A-tailing, adapter ligation, and library enrichment PCR. In addition, during the post-enrichment SPRI cleanup, elution volume was reduced to 30 μL to maximize library concentration, and a vortexing step was added to maximize the amount of template eluted.

#### In-solution hybrid selection

After library construction, libraries were pooled into groups of up to 96 samples. Hybridization and capture were performed using the relevant components of Illumina’s Nextera Exome Kit and following the manufacturer’s suggested protocol, with the following exceptions. First, all libraries within a library construction plate were pooled prior to hybridization. Second, the Midi plate from Illumina’s Nextera Exome Kit was replaced with a skirted PCR plate to facilitate automation. All hybridization and capture steps were automated on the Agilent Bravo liquid handling system.

#### Preparation of libraries for cluster amplification and sequencing

After post-capture enrichment, library pools were quantified using qPCR (automated assay on the Agilent Bravo) using a kit purchased from KAPA Biosystems with probes specific to the ends of the adapters. Based on qPCR quantification, libraries were normalized to 2 nM.

#### Cluster amplification and sequencing

Cluster amplification of DNA libraries was performed according to the manufacturer’s protocol (Illumina) using exclusion amplification chemistry and flowcells. Flowcells were sequenced utilizing sequencing-by-synthesis chemistry. The flow cells were then analyzed using RTA v.2.7.3 or later. Each pool of whole-exome libraries was sequenced on paired 76 cycle runs with two 8 cycle index reads across the number of lanes needed to meet coverage for all libraries in the pool. Pooled libraries were run on HiSeq 4000 paired-end runs to achieve a minimum of 150x on target coverage per each sample library. The raw Illumina sequence data were demultiplexed and converted to fastq files; adapter and low-quality sequences were trimmed. The raw reads were mapped to the hg38 human reference genome, and the validated BAMs were used for downstream analysis and variant calling.

#### Quality assurance and quality control of RNA analytes

All RNA analytes were assayed for RNA integrity, concentration, and fragment size. Samples for total RNA-seq were quantified on a TapeStation system (Agilent, Inc. Santa Clara, CA). Samples with RINs >8.0 were considered high quality.

#### Total RNA-seq library construction

Total RNA-seq library construction was performed from the RNA samples using the TruSeq Stranded RNA Sample Preparation Kit and bar-coded with individual tags following the manufacturer’s instructions (Illumina, Inc. San Diego, CA). Libraries were prepared on an Agilent Bravo Automated Liquid Handling System. Quality control was performed at every step and the libraries were quantified using the TapeStation system.

#### Total RNA sequencing

Indexed libraries were prepared and run on HiSeq 4000 paired-end 75 base pairs to generate a minimum of 120 million reads per sample library with a target of greater than 90% mapped reads. Typically, these were pools of four samples. The raw Illumina sequence data were demultiplexed and converted to FASTQ files, and adapter and low-quality sequences were quantified. Samples were then assessed for quality by mapping reads to the hg38 human genome reference, estimating the total number of reads that mapped, amount of RNA mapping to coding regions, amount of rRNA in sample, number of genes expressed, and relative expression of housekeeping genes. Samples passing this QA/QC were then clustered with other expression data from similar and distinct tumor types to confirm expected expression patterns. Atypical samples were then SNP typed from the RNA data to confirm the source analyte. FASTQ files of all reads were then uploaded to the GDC repository.

#### Single-nuclei RNA library preparation and sequencing

About 20–30 mg of cryopulverized powder from ccRCC specimens was resuspended in Lysis buffer (10 mM Tris-HCl (pH 7.4); 10 mM NaCl; 3 mM MgCl2; and 0.1% NP-40). This suspension was pipetted gently 6–8 times, incubated on ice for 30 s, and pipetted again 4-6 times. The lysate containing free nuclei was filtered through a 40 μm cell strainer. We washed the filter with 1 mL Wash and Resuspension buffer (1X PBS +2% BSA +0.2 U/μL RNase inhibitor) and combined the flow through with the original filtrate. After 6-min centrifugation at 500 x g and 4°C, the nuclei pellet was resuspended in 500 μL of Wash and Resuspension buffer. After staining by DRAQ5, the nuclei were further purified by Fluorescence-Activated Cell Sorting (FACS). FACS-purified nuclei were centrifuged again and resuspended in a small volume (about 30 μL). After counting and microscopic inspection of nuclei quality, the nuclei preparation was diluted to about 1,000 nuclei/μL.

About 20,000 nuclei were used for single-nuclei RNA sequencing (snRNA seq) by the 10X Chromium platform. We loaded the single nuclei onto a Chromium Chip B Single Cell Kit, 48 rxns (10x Genomics, PN-1000073), and processed them through the Chromium Controller to generate GEMs (Gel Beads in Emulsion). We then prepared the sequencing libraries with the Chromium Single Cell 3′ GEM, Library & Gel Bead Kit v3, 16 rxns (10x Genomics, PN 1000075) following the manufacturer’s protocol. Sequencing was performed on an Illumina NovaSeq 6000 S4 flow cell. The libraries were pooled and sequenced using the XP workflow according to the manufacturer’s protocol with a 28 × 8 × 98bp sequencing recipe. The resulting sequencing files were available as FASTQs per sample after demultiplexing.

#### Illumina Infinium methylationEPIC beadchip array

The MethylationEPIC array uses an 8-sample version of the Illumina Beadchip capturing >850,000 DNA methylation sites per sample. 250 ng of DNA was used for the bisulfite conversation using Infinium MethylationEPIC BeadChip Kit. The EPIC array includes sample plating, bisulfite conversion, and methylation array processing. After scanning, the data was processed through an automated genotype calling pipeline. Data generated consisted of raw idats and a sample sheet.

#### Sample processing for protein extraction and tryptic digestion

All samples for the current study were prospectively collected as described above and processed for mass spectrometric (MS) analysis at Johns Hopkins University. Tissue lysis and downstream sample preparation for global proteomic, phosphoproteomic and glycoproteomic analysis were carried out as previously described.^24^^,^^25^^,^^124^ Each of cryopulverized renal tumor tissues or NATs were homogenized separately in an appropriate volume of lysis buffer (8 M urea, 75 mM NaCl, 50 mM Tris, pH 8.0, 1 mM EDTA, 2 μg/mL aprotinin, 10 μg/mL leupeptin, 1 mM PMSF, 10 mM NaF, Phosphatase Inhibitor Cocktail 2 and Phosphatase Inhibitor Cocktail 3 [1:100 dilution], and 20 μM PUGNAc) by repeated vortexing.

Proteins in the lysates were clarified by centrifugation at 20,000 x g for 10 min at 4C, and protein concentrations were determined by BCA assay (Pierce). The proteins were diluted to a final concentration of 8 mg/mL with a lysis buffer for the downstream reduction, alkylation and digestion. 1.2 mg of protein was reduced with 5 mM dithiothreitol (DTT) for 1 h at 37 C and subsequently alkylated with 10 mM iodoacetamide for 45 min at RT (room temperature) in the dark. Samples were then diluted by 1:4 with 50 mM Tris-HCl (pH 8.0) and subjected to proteolytic digestion with LysC (Wako Chemicals, at 1:50 enzyme-to-substrate weight ratio for 2 h incubation at RT) followed by the addition of sequencing-grade modified trypsin (Promega, at a 1:50 enzyme-to-substrate weight ratio for overnight incubation at RT). The digested samples were then acidified with 50% formic acid (FA, Fisher Chemicals) to pH < 3. Tryptic peptides were desalted on reversed-phase C18 SPE columns (Waters) and dried using a Speed-Vac (Thermo Scientific).

#### TMT labeling of peptides

Tandem-mass-tag (TMT) quantitation utilizes reporter ion intensities to determine protein abundance and facilitate quantitative proteomic analysis.^125^ The samples from the discovery cohort were labeled with TMT-10plex as described in the ccRCC discovery paper,^24^ while the samples from the non-ccRCC cohort were labeled with TMT-11plex reagents (Thermo Fisher Scientific). 70 non-ccRCC samples were co-randomized to 7 TMT 11-plex sets. The sample-to-TMT channel mapping is available in the PDC portal (https://proteomic.datacommons.cancer.gov/). 300ug desalted peptides from each non-ccRCC and NAT sample were dissolved in 120 μL of 100 mM HEPES, pH 8.5 solution. 5mg TMT reagent was dissolved in 500 μL of anhydrous acetonitrile, and 45 μL of each TMT reagent was added to the corresponding aliquot of peptides. After 1 h incubation at RT, the reaction was quenched by incubation with 5% hydroxylamine at RT for 15 min. The reference sample used in the ccRCC discovery cohort study^24^ was included in all TMT 11-plexes as a reference channel in the non-ccRCC cohort study, labeled with the TMT-131 reagent. Following labeling, peptides were mixed according to the sample-to-TMT channel mapping, concentrated and desalted on reversed-phase C18 SPE columns (Waters), and dried using a Speed-Vac (Thermo Scientific).

#### Peptide fractionation by basic reversed-phase liquid chromatography

To reduce the likelihood of peptides co-isolating and co-fragmenting in these highly complex samples, we employed extensive, high-resolution fractionation via basic reversed-phase liquid chromatography (bRPLC). The desalted and dried peptides from each TMT set were reconstituted in 900 mL of 5 mM ammonium formate (pH 10) and 2% acetonitrile (ACN) and loaded onto a 4.6 mm × 250 mm RP Zorbax 300 A Extend-C18 column with 3.5 μm size beads (Agilent). Peptides were separated at a flow-rate of 1 mL/min using an Agilent 1200 Series HPLC instrument with Solvent A (2% ACN, 5 mM ammonium formate, pH 10) and a non-linear gradient of Solvent B (90% ACN, 5 mM ammonium formate, pH 10) as follows: 0% Solvent B (7 min), 0%–16% Solvent B (6 min), 16%–40% Solvent B (60 min), 40%–44% Solvent B (4 min), 44%–60% Solvent B (5 min), and holding at 60% Solvent B for 14 min. Collected fractions were concatenated into 24 fractions by combining four fractions that are 24 fractions apart as described previously^25^; a 5% aliquot of each of the 24 fractions was used for global proteomic analysis, dried in a Speed-Vac, and resuspended in 3% ACN/0.1% formic acid prior to ESI-LC-MS/MS analysis. The remaining sample was utilized for phosphopeptide enrichment.

#### Enrichment of phosphopeptides by Fe-IMAC

The remaining 95% of the sample was further concatenated into 12 fractions before being subjected to phosphopeptide enrichment using immobilized metal affinity chromatography (IMAC) as previously described.^25^ In brief, Ni-NTA agarose beads (Qiagen) were conditioned and incubated with 10mM FeCl3 to prepare Fe3+-NTA agarose beads. Dried peptides from each fraction were reconstituted in 80% ACN/0.1% trifluoroacetic acid and incubated with 10 μL of the Fe3+-IMAC beads for 30 min. Samples were then centrifuged at 1000∗g for 1 min to collect the beads coupled with phophopeptides, and the supernatant containing unbound peptides was removed for the subsequent glycopeptides enrichment (Cao, PDA paper, cell, 2021). The beads were resuspended with 80% ACN/0.1% trifluoroacetic acid and then transferred onto equilibrated C-18 Stage Tips. Tips were washed twice with 80% ACN/0.1% trifluoroacetic acid followed by 1% formic acid.

The flowthroughs were collected and combined with the supernatants for subsequent glycopeptides enrichments. Phosphopeptides were eluted from the Fe3+-IMAC beads onto the C-18 Stage Tips with 70 μL of 500 mM dibasic potassium phosphate, pH 7.0 three times. C-18 Stage Tips were then washed twice with 1% formic acid to remove salts, followed by elution of the phosphopeptides from the C-18 Stage Tips with 50% ACN/0.1% formic acid twice. Eluted phosphopeptides were dried down and resuspended in 3% ACN/0.1% formic acid prior to ESI-LC-MS/MS analysis.

#### Enrichment of intact glycopeptides by MAX columns

All unbound peptides from phosphopeptide enrichment were desalted on reversed phase C18 SPE column (Waters). The glycopeptides were enriched with OASIS MAX solid-phase extraction (Waters). The MAX cartridge was conditioned with 3 × 1 mL ACN, then 3 × 1 mL of 100 mM triethylammonium acetate buffer, followed by 3 × 1 mL of water, and finally 3 × 1 mL of 95% ACN (1% TFA). The peptides were loaded twice. The cartridge was washed with 4 × 1 mL of 95% ACN (1% TFA) to remove non-glycosylated peptides. The glycopeptide fraction was eluted with 50% ACN (0.1% TFA), dried down, and reconstituted in 3% ACN, 0.1% FA prior to ESI-LC-MS/MS analysis.

#### ESI-LC-MS/MS for global proteome, phosphoproteome, and glycoproteome analysis

The TMT-labeled global proteome, phosphoproteome, and glycoproteome fractions were analyzed using Orbitrap Fusion Lumos mass spectrometer (Thermo Scientific). Approximately 0.8 μg of peptides were separated on an in-house packed 28 cm × 75 mm diameter C18 column (1.9 mm Reprosil-Pur C18-AQ beads (Dr. Maisch GmbH); Picofrit 10 mm opening (New Objective)) lined up with an Easy nLC 1200 UHPLC system (Thermo Scientific). The column was heated to 50°C using a column heater (Phoenix-ST). The flow rate was set at 200 nL/min. Buffer A and B were 3% ACN (0.1% FA) and 90% ACN (0.1% FA), respectively. The peptides were separated with a 6%–30% B gradient in 84 min. Peptides were eluted from the column and nanosprayed directly into the mass spectrometer. The mass spectrometer was operated in a data-dependent mode.

Parameters for global proteomic and phosphoproteomic samples were set as follows: MS1 resolution - 60,000, mass range – 350 to 1800 m/z, RF Lens – 30%, AGC Target – 4.0e5, Max injection time – 50 ms, charge state include – 2–6, dynamic exclusion – 45 s. The cycle time was set to 2 s, and within this 2 s the most abundant ions per scan were selected for MS/MS in the orbitrap. MS2 resolution – 50,000, high-energy collision dissociation activation energy (HCD) – 34, isolation width (m/z) – 0.7, AGC Target – 2.0e5, Max injection time – 100 ms. Parameters for glycoproteomic samples were set as follows: MS1 resolution - 60,000, mass range – 500 to 2000 m/z, RF Lens – 30%, AGC Target – 5.0e5, Max injection time – 50 ms, charge state include – 2–6, dynamic exclusion – 45 s. The cycle time was set to 2 s, and within this 2 s the most abundant ions per scan were selected for MS/MS in the orbitrap. MS2 resolution – 50,000, high-energy collision dissociation activation energy (HCD) – 35, isolation width (m/z) – 0.7, AGC Target – 1.0e5, Max injection time – 100 ms.

#### Metabolomic acquisition

To extract metabolites, a solution consisting of 80% (v/v) mass spectrometry-grade methanol and 20% (v/v) mass spectrometry-grade water were used to extract the metabolites from the tissue samples as described previously.^126^^,^^127^^,^^128^ The metabolite samples then underwent speed vacuum processing to evaporate the methanol and lyophilization to remove the water. The dried metabolites were re-suspended in a solution consisting of 50% (v/v) acetonitrile and 50% (v/v) mass spectrometry-grade water before data acquisition. Data acquisition was performed using a Vanquish ultra-performance liquid chromatography (UPLC) system and a Thermo Scientific Q Exactive Plus Orbitrap Mass Spectrometer.

The samples were kept at 4°C inside the Vanquish UPLC auto-sampler. The injection volume for each sample was 2 μL. A Discovery HSF5 reverse phase HPLC column (Sigma) kept at 35°C with a guard column was used for reverse-phase chromatography. The mobile aqueous phase was mass spectrometry-grade water containing 0.1% formic acid, while the mobile organic phase was acetonitrile containing 0.1% formic acid. Mass calibration was performed prior to data acquisition to ensure the sensitivity and accuracy of the system. The total run time for each sample was 15 min, for which 11 min was used for data acquisition. Full MS data were acquired to quantify the metabolites while Full MS/ddMS2 data were also acquired to identify the metabolites based on fragmentation matching.

### Quantification and statistical analysis

#### Somatic mutation calling

WES reads were aligned FASTQ files to the GRCh38 references, including alternate haplotypes. Variant calling was performed using VarDict (germline & somatic) and Strelka2 (somatic). Variant callers were run with default settings, but custom filters were applied. Strelka was used to generate the primary somatic call-set. Variants called by Strelka had to be either (FILTER = = ”PASS”) or meet the following threshold criteria: allele frequency in the tumor >0.05, allele frequency in the normal <0.01, at least five variant reads, depth in normal >50, Somatic Evidence Score (EVS) > 90th percentile of overall EVS distribution. These calls were supplemented by variants called confidently (FILTER = = ”PASS” and manual review) by VarDict^129^ in genes recurrently mutated in ccRCC: VHL, PBRM1, BAP1, SETD2, KDM5C, PTEN, MTOR, TP53, PIK3CA, ARID1A, STAG2, KDM6A, KMT2C, KMT2D. This strategy improved sensitivity in ccRCC-mutated genes without sacrificing the accuracy of variant calls genome wide.

#### Gene fusion

Detection of gene fusions was performed using the CODAC algorithm as previously described.^130^^,^^131^ Briefly, CODAC implements detection of genic and intergenic gene fusions based on both split- and discordant-reads as detected through chimeric read alignment using STAR.^132^ To maximize sensitivity, STAR is run separately using optimized settings in single-end and paired-end mode for overlapping and non-overlapping read pairs, respectively. The resulting alignments are merged, resulting in candidate fusion junctions identified from the STAR alignments are evaluated based on alignment properties to identify false-positive calls, including incorrect mappings, reference errors/differences, non-genetic sources, such as circRNAs. The resulting call-set is further filtered against a manually-curated database of recurrent artifacts. STAR settings:

--alignIntronMax 150000 \

--alignMatesGapMax 150000 \

--chimSegmentMin 10 \

--chimJunctionOverhangMin 1 \

--chimScoreSeparation 0 \

--chimScoreJunctionNonGTAG 0 \

--chimScoreDropMax 1000 \

--chimScoreMin 1 \

#### Copy number estimation

Copy-number analysis was performed jointly leveraging both whole-genome sequencing (WGS) and whole-exome sequencing data of the tumor and germline DNA. To perform the analysis, we used CNVEX (https://github.com/mctp/cnvex), a comprehensive copy number analysis tool that has been used previously in our ccRCC studies.^24^^,^^36^ CNVEX uses whole-genome aligned reads to estimate coverage within fixed genomic intervals and whole exome variant calls to compute B-allele frequencies (BAFs) at variant positions (called by Sentieon DNAscope algorithm). Coverages were computed in 10kb bins, and the resulting log coverage ratios between tumor and normal samples were adjusted for GC bias using weighted LOESS smoothing across mappable and non-blacklisted genomic intervals within the GC range 0.3–0.7, with a span of 0.5 (the target and configuration files are provided with CNVEX). The adjusted log coverage ratios (LR) and BAFs were jointly segmented by a custom algorithm based on Circular Binary Segmentation (CBS). Alternative probabilistic algorithms were implemented in CNVEX, including algorithms based on recursive binary segmentation (RBS), as implemented in the R-package jointseg.^133^ For the CBS-based algorithm, first LR and mirrored BAF were independently segmented using CBS (parameters alpha = 0.01, trim = 0.025) and all candidate breakpoints were collected. The resulting segmentation track was iteratively ‘‘pruned’’ by merging segments that had similar LR and BAFs, short lengths, were rich in blacklisted regions, and had a high coverage variation in coverage among whole cohort germline samples. For the RBS- and DP-based algorithms, joint-break-points were ‘‘pruned’’ using a statistical model selection method (https://hal.inria.fr/inria-00071847). For the final set of CNV segments, we chose the CBS-based results as they did not require specifying a prior number of expected segments (K) per chromosome arm, were robust to unequal variances between the LR and BAF tracks and provided empirically the best fit to the underlying data. The resulting segmented copy-number profiles were then subject to the joint inference of tumor purity and ploidy and absolute copy number state, implemented in CNVEX, which is most similar to the mathematical formalism of ABSOLUTE^134^^,178^ and PureCN^135^ (http://bioconductor.org/packages/PureCN/).

Briefly, the algorithm inputs the observed log-ratios (of 10kb bins) and BAFs of individual SNPs. LRs and BAFs are assigned to their joint segments and their likelihood is determined given a particular purity, ploidy, absolute segment copy number, and the number of minor alleles. To identify candidate combinations with a high likelihood, we followed a multi-step optimization procedure that includes grid-search (across purity-ploidy combinations), greedy optimization of absolute copy numbers, and maximum-likelihood inferences of minor allele counts. Following optimization, CNVEX ranks candidate solutions. Because the copy-number inference problem can have multiple equally likely solutions, further biological insights are necessary to choose the most parsimonious result. The solutions have been reviewed by independent analysts following a set of guidelines. Solutions implying whole genome duplication must be supported by at least one large segment that cannot be explained by a low-ploidy solution, inferred purity must be consistent with the variant-allele-frequencies of somatic mutations, and large homozygous segments are not allowed.

In parallel, we used BIC-seq2,^136^ a read-depth-based CNV calling algorithm to detect somatic copy number variation (CNVs) from the WGS data of tumors. Briefly, BIC-seq2 divides genomic regions into disjoint bins and counts uniquely aligned reads in each bin. Then, it combines neighboring bins into genomic segments with similar copy numbers iteratively based on Bayesian Information Criteria (BIC), a statistical criterion measuring both the fitness and complexity of a statistical model. We used paired-sample CNV calling that takes a pair of samples as input and detects genomic regions with different copy numbers between the two samples. We used a bin size of ∼100 bp and a lambda of 3 (a smoothing parameter for CNV segmentation). We recommend calling segments as copy gain or loss when their log2 copy ratios were larger than 0.2 or smaller than −0.2, respectively (according to the BIC-seq publication).

#### RNA-seq data processing

Transcriptomic data were analyzed as described previously,^130^ using the Clinical RNA-seq Pipeline (CRISP) (https://github.com/mcieslik-mctp/crisp-build) of TPO. Briefly, raw sequencing data were trimmed, merged using BBMap, and aligned to GRCh38 using STAR.^132^ The resulting BAM files were analyzed for expression using feature counts against a transcriptomic reference based on Gencode 34. The resulting gene-level counts for protein-coding genes were transformed into FPKMs using edgeR.^137^

#### snRNA-seq data processing

Read alignment and quantification were conducted with Cell Ranger (v3.1.0) and pre-mRNA reference genome created based on 10X pre-built reference genome (GRCh38). Specifically, for each sample, we obtained the unfiltered feature-barcode matrix per sample by passing the demultiplexed FASTQs to Cell Ranger v3.1.0 ‘count’ command using default parameters, and a customized pre-mRNA GRCh38 genome reference was built to capture both exonic and intronic reads. The customized genome reference modified the transcript annotation from the 10x Genomics pre-built human genome ref. 3.0.0 (GRCh38 and Ensembl 93). Starting with unfiltered count matrix, non-empty barcodes were identified with DropletUtils^138^^,^^139^ correction for potential background RNA contamination was performed with SoupX.^140^ Cells with outlier numbers of total UMIs/genes and mitochondrial gene fraction were identified using scatter and discarded. For total UMI/genes, values were 3 median-absolute-deviations or MADs higher or lower from median were considered outliers; for mitochondrial fractions, values were 3 MADs higher than median were considered outliers. Subsequently, mitochondrial genes were removed from the entire count matrix as they probably represented contamination from cytoplasm during nuclei preparation.

#### Identification and quantification of global proteome and phosphoproteome

Raw mass spectrometry files were converted into open mzML format using the msconvert utility of the Proteowizard software suite, and analyzed using FragPipe computational platform (https://fragpipe.nesvilab.org/) using the TMT11-bridge workflow where the common ccRCC pool sample was used as bridge to link the two cohort. (the 11th channel was removed later in the discovery cohort). MS/MS spectra were searched using the database search tool MSFragger v3.4^110^ against a harmonized Homo sapiens GENCODE34 protein sequence database appended with an equal number of decoy sequences. Whole cell lysate MS/MS spectra were searched using a precursor-ion mass tolerance of 20 ppm and allowing C12/C13 isotope errors −1/0/1/2/3. Mass calibration and parameter optimization were enabled. Cysteine carbamidomethylation (+57.0215) and lysine TMT labeling (+229.1629) were specified as fixed modifications, and methionine oxidation (+15.9949), N-terminal protein acetylation (+42.0106), and TMT labeling of peptide N terminus and serine residues were specified as variable modifications.

For the analysis of phosphopeptide enriched data, the set of variable modifications also included phosphorylation (+79.9663) of serine, threonine, and tyrosine residues. The search was restricted to tryptic peptides, allowing up to two missed cleavage sites. Peptide to spectrum matches (PSMs) were further processed using Percolator^141^ to compute the posterior error probability, which was then converted to posterior probability of correct identification for each PSM. The resulting files from Percolator were converted to pep.xml format, and with the phosphopeptide-enriched dataset, pep.xml files were additionally processed using PTMProphet^142^ to localize the phosphorylation sites. The resulting files were then processed together to assemble peptides into proteins (protein inference) using ProteinProphet^143^ run via the Philosopher toolkit v4.0.1^109^ to create a combined set of high confidence protein groups. The combined prot.xml file and the individual PSM lists for each TMT experiment were further processed using the Philosopher filter command as follows.

Each peptide was assigned either as a unique peptide to a particular protein group or assigned as a razor peptide to a single protein group that had the most peptide evidence. The protein groups assembled by Percolator were filtered to 1% protein-level False Discovery Rate (FDR) using the target-decoy strategy and the best peptide approach (allowing both unique and razor peptides). The PSM lists were filtered using a sequential FDR strategy, keeping only those PSMs that passed 1% PSM-level FDR filter and mapped to proteins that also passed the global 1% protein-level FDR filter. In addition, for all PSMs corresponding to a TMT-labeled peptide, reporter ion intensities were extracted from the MS/MS scans (using 0.002 Da window) using Philosopher and the precursor ion purity scores were calculated using the intensity of the sequenced precursor ion and that of other interfering ions observed in MS1 data (within a 0.7 Da isolation window). The PSM output files were further processed using TMT-Integrator v3.2.0 to generate summary reports at the gene-level and modification-site level.

TMT-Integrator^112^(https://github.com/Nesvilab/TMT-Integrator) used the PSM tables generated by the Philosopher pipeline as described above as input and created integrated reports with quantification across all samples. First, PSMs were filtered to remove all entries that did not pass at least one of the quality filters, such as PSMs with (a) no TMT label; (b) precursor-ion purity less than 50%; (c) summed reporter ion intensity (across all channels) in the lower 5% percentile of all PSMs in the corresponding PSM.tsv file (2.5% for phosphopeptide enriched data); (d) peptides without phosphorylation (for phosphopeptide enriched data). In the case of redundant PSMs (i.e., multiple PSMs in the same MS run sample corresponding to the same peptide ion), only the single PSM with the highest summed TMT intensity was retained for subsequent analysis. Both unique and razor peptides were used for quantification, while PSMs mapping to common external contaminant proteins (that were included in the searched protein sequence database) were excluded.

Next, for each PSM the intensity in each TMT channel was converted into a log2-based ratio to the reference channel. The PSMs were grouped to the gene level, and the gene ratios were computed as the median of the corresponding PSM ratios after outlier removal. Ratios were then converted back to absolute intensity in each sample by using the reference gene intensity estimated, using the sum of all MS2 reporter ions from all corresponding PSMs. To generate peptide-level and site-level tables, additional post-processing was applied to generate all non-conflicting phosphosite configurations using a strategy similar to that described in Huang et al.^144^ In doing so, confidently localized sites were defined as sites with PTMProphet localization probability of 0.75 or higher. The same peptide sequences but with different site configurations, i.e., different site localization configurations or peptides with unlocalized sites, were retained as separate entries in the site-level tables. In the peptide-level tables, different site-level configurations were combined into a single peptide-level index, grouping PSMs with all site configurations together if they corresponded to the same peptide sequence. The tutorial describing all steps of the analysis, including specific input parameter files, command-line option, and all software tools necessary to replicate the results are available at https://github.com/Nesvilab.

#### Quantification of intact glycopeptides and glycosite localization

Raw files of the glyco-enriched samples and phospho-enriched samples were searched for N-linked glycopeptides via MSFragger^110^ (version 3.3) and Philosopher^109^ (version 4.0). Parameters were as described for whole proteome search, except as follows. C12/C13 isotope errors of 0/1/2 were allowed, and methionine oxidation (+15.99491) was the only specified variable modification for glyco-enriched samples. Phosphorylation of serine, threonine, and tyrosine (+79.96633) was specified for phospho-enriched samples. “Nglycan” search mode was used, restricting glycosylation sites to the consensus sequon N-X-S/T, where X is any residue other than proline. A customized human N-glycan database which contained 252 compositions.^144^ Diagnostic ion filtering for glycopeptide spectra was enabled with a minimum intensity threshold of 10% and the following list of oxonium ions considered: 204.086646, 186.076086, 168.065526, 366.139466, 144.0656, 138.055, 512.197375, 292.1026925, 274.0921325, 657.2349, 243.026426, 405.079246, 485.045576, 308.09761. Glycan Y ions of 203.07937 and 406.15874 were included in search, along with a remainder mass of 203.07937 on peptide b and y ions (“b∼/y∼” ions). PSMs were further processed with PeptideProphet,^145^ using the extended mass model with a mass width of 4000, as described in Polasky et al.^62^ Protein inference, FDR filtering, and reporter ion intensity extraction were accomplished as in the whole proteome search.

Glycan assignment and glycan-specific FDR filtering was subsequently performed in PTM-Shepherd as previously described.^63^ Briefly, possible glycan compositions given the observed delta mass recorded by MSFragger were scored using the glycan fragment ions observed in the spectrum and filtered to 1% FDR by comparison to spectrum-based decoy glycans. Default settings were used except for consideration of a single ammonium adduct on possible glycan compositions. PTM-Shepherd assigned glycan compositions and confidence scores were written back to the PSM tables. The PSM output files were then processed with TMT-Integrator^112^ v3.1.2 to generate summary reports at the gene, protein, peptide, site, and “multi-mass” levels from glycopeptide spectra. Multi-mass refers to the combination of glycan and site, i.e., each distinct glycan identified at a given site generates a separate entry. The PSM filtering and summarization process was the same as for whole proteome searches, with the exception of restricting the PSMs considered to those of glycopeptides and using the MSFragger-reported localization of the glycosite within identified peptides rather than PTMProphet.

#### Identification and quantification of metabolomic data

Acquired data were analyzed first using Thermo Scientific Compound Discoverer software. The chromatographic peaks were integrated to obtain raw intensities of metabolites. Compounds with definite peaks and names in the software were selected. The data were then filtered based on the following criteria: m/z Cloud score greater than 60 (good fragmentation matching with compounds in the m/z Cloud database) or mass list match (mass lists include common pathways such as glycolysis, pentose phosphate pathway, hexosamine, and sialic acid pathway, purine and pyrimidine synthesis, and amino acid metabolism) and intensity >10000. Thermo Scientific TraceFinder software was then used to quantify compounds in common pathways not found using Compound Discoverer where the retention time (RT) was determined using Freestyle software based on mass accuracy and fragmentation match. The data from Thermo Scientific Compound Discoverer and TraceFinder software were combined to generate the final list of compounds.

#### Proteomic data normalization and imputation

With the output from FragPipe, TMT-Integrator reports, we first filter out contaminated genes and samples, map ENSEMBL ID to gene symbols, and remove duplicated genes and samples by using the average quantification. For global proteomics and phosphoproteomics data, we performed data imputation to support some downstream analysis such as NMF clustering. Genes with missing values more than 50% are filtered out. Separately in the discovery cohort and non-ccRCC cohort, we correct the batch effect caused by potential uneven TMT plexing with Combat after imputing missing values in the remaining genes with KNN. Then we join the two datasets together using normal samples as control. We replace imputed values with NA and run DreamAI imputation with default parameter setting.

#### PTM proteomics data normalization

With TMT-Integrator’s ratio reports on global proteome and PTM (phosphorylation and glycosylation (protein level)) datasets, we build a simple linear regression model with all the samples’ global protein ratio as predictor and their respective PTM ratio data as response. After the model is fitted, the residual values are taken as normalized PTM intensity.

#### Principal component analysis

We performed PCA on 150 tumor samples including (103 ccRCC, 15 RO, 13 pRCC, 3 chRCC, 2 AML, 2 ESCRCC, 1 BHD, 1 MEST, 1 MTOR mutated RCC, 1 TRCC, 7 MDTH) and 101 normal adjacent (NAT) samples to illustrate the gene expression (RNA-seq, 89 NAT), global proteomic, phosphoproteomic, glycoproteomic difference between tumor and NAT samples. Due to sample availability, in metabolomics data analysis, only 28 tumors (8 RO, 8 pRCC, 2 AML, 1 chRCC, 1 ESCRCC, 1 BHD, 1 MEST, 1 MTOR mutated RCC and 5 unRCC) and 7 NATs went through PCA (Figure S1D). R function prcomp was employed to calculate loadings on each principal component. R function fviz from library “factoextra” was employed to visualize the results in 2D, ellipse of subtype groups were added with specifying parameter addEllipses = T and ellipse.level = 0.5.

#### Tumor versus normal and between-subtypes Differential expression/proteomic analysis

TMT-based global proteomics data without missing value filtering were used to perform differential proteome analysis between tumor and normal samples, as well as between different subtypes. R package limma^90^ was used to fit a linear regression model between sample groups for proteomics data in log2 scale. Tumor purity adjustment is achieved differently in different types of comparisons. In tumor subtype comparisons, tumor purity is added as a co-variable in the regression model. In tumor versus normal comparisons, tumor purity is the only variable in the regression model, as tumor purity for normal tissues is zero. After model fitting, the regression coefficient is the fold change in log2 scale between comparison groups (mean difference between two groups) and the *p* value and q value associated with the moderated t statistic (p.mod and q.mod) calculated with the ebayes function are the resultant significance measurement. RNA differential expression analysis was done similarly as proteomic analysis on TPM normalized data.

Nonnegative Matrix Factorization (NMF) clustering and Heatmap Analysis.

Filtered RNA (TPM) data based on raw read counts, imputed global proteomics ratio data, and imputed phosphoproteomics ratio data were supplied to SignatureAnalyzer (https://github.com/getzlab/SignatureAnalyzer) to perform automatic relevance determination (ARD) NMF clustering.^30^ Clustering results with immune deconvolution result, mutation information, copy number variations are visualized with heatmaps through R library ComplexHeatmap^146^ (Figure 1A).

#### Immune deconvolution

To estimate the fraction of different cell types in the tissue microenvironment, we performed a multi-omic based deconvolution integrating global proteomic and RNA-seq data via BayesDebulk.^119^ Only samples with both gene expression and global proteomic measurements were considered. Protein abundance was imputed as described above before being inputted to BayesDebulk. To perform the deconvolution, BayesDeBulk requires a list of cell-type specific markers for each cell type. For immune cells, such list was derived from the LM22 signature matrix^147^ in a similar fashion as in Petralia et al.^119^ For this analysis, an aggregated version of the LM22 signature matrix was utilized. Specifically, we averaged the LM22 values mapping to different types of CD4 T Cells (e.g., Memory T Cells, Naive T Cells) to create a gene signature for CD4 T Cells. The same strategy was utilized for Dendritic cells, Natural Killers cells, Mast Cells and B Cells. For each pair of cell types, we considered a marker to be upregulated in the first cell type compared to the other cell type, if the corresponding value of the LM22 matrix for the first cell type was greater than 1,000 and 5 times the value of the other cell type. For Endothelial-PLVAP, Endothelial-ACKR1, Pericytes and vSMC, we used marker signatures from a previous ccRCC single-cell RNA-seq study.^36^ To derive these signatures, differential expression between different single-cell clusters was performed and only markers significant at 10% FDR and a log fold change greater than 1 were considered as cell-type specific markers.

Finally, we considered Macrophage A and Macrophage B signatures from Zhang et al.^36^ As common markers for Macrophage A and B we used *C1QA*, *C1QB*, *C1QC*, *MS4A6A*, *LYZ*, *TYROBP*, *FCGR2A, FCER1G, AIF1, CD14, CD68*; as markers specific of Macrophages A we considered: *CXCL8, CXCL2, CCL4, CCL3, CCL4L2, CXCL3, CCL3L3, CCL20, NFKB1, IL1B*; while for Macrophage B the following cell-type specific markers were considered: *CTSL, LGMN, ASAH1, LIPA, CTSD, LAMP1*. BayesDeBulk was estimated via 10,000 Monte Carlo Markov Chain (MCMC) iterations. Cell-type fractions were estimated as the mean across MCMC iterations after discarding a burn-in of 1,000 iterations. Once estimated, cell-type fractions for each patient were standardized to sum to the total fraction of immune/stromal cells in the tissue microenvironment. The total fraction of immune/stromal cells was computed as one minus the tissue purity inferred from gene expression data. The estimation of the purity was performed via TSNet.^148^

#### Immune/methylation subtype clustering

Immune deconvolution results and methylation data were subjected to a consensus clustering algorithm to identify subtypes within tumors. Percentages of immune cells calculated by BayesDebulk were used for immune subtyping. For methylation subtyping, beta values from the methylation array harmonization workflow based on SeSAMe^149^ were downloaded from CPTAC DCC and GDC. To avoid methylation probes of ccRCC from overrepresentation, top 4000 most variable probes with less than 50% missing values were selected independently for ccRCC-Discovery cohort and non-ccRCC cohort. Selected probes were then combined with remaining missing values imputed by the mean of the corresponding probe. Consensus clustering was performed via CancerSubtypes^113^ with following parameters: maxK = 10, reps = 1000, pItem = 0.8, pFeature = 1, clusterAlg = "km", distance = "euclidean". Numbers of clusters were chosen based on the delta area plot of the consensus CDF.

#### High versus low wGII groups determination

To measure overall instability, we used an already published measure of Weighted Genome Instability Index (wGII).^150^ WGII was measured as the proportion of each chromosome which has a different copy number compared to the baseline copy number of the sample. Then the average of scores for each chromosome was calculated, weighted by the length of the chromosome such that each chromosome has the same contribution to the overall instability score.

To validate our results, wGII were also calculated for TCGA kidney cohorts (KIRC, KIRP, KICH). All the required information regarding the segmentation, absolute copy numbers and purity values were acquired through TCGA PanCanAtlas GDC portal.^151^ The cutoff of high or low wGII grouping was determined by finding the cutoff that minimizes the *p*-value of survival differences of the two groups using a cox-regression modeling (cutoff = 0.32), using the TCGA-KIRP cohort. In practice, to make sure each group has enough samples, we used the cutoff of 0.3 which yields a more balanced populated grouping, but still a significant *p*-value.

#### Dimension reduction in single nucleus RNA-seq and cell type assignment

Following data processing all the sample libraries passed various QC measures including floating RNA contamination that was estimated by SOUPX median 6% (range 1.2–17%). Data from 79,673 nuclei from 8 samples (median 10,592) were used in integrative analysis with previously published snRNA-seq data from benign kidney samples. Cell type annotations were rendered by examining biomarker expression patterns identified based on previous single cell RNA-seq and single nucleus RNA-seq data.^35^^,^^36^

Downstream analyses were performed with Seurat^152^ v4.1. Filtered count matrix was normalized to 10000 UMI per cell and natural-log transformed, top 2000 highly variable genes (HVGs) were identified by modeling the mean-variance relationship, PCA was then performed on scaled and centered matrix (including HVGs only); finally cells were projected into a 2-D map with UMAP^153^ using the first 30 PCs. Clusters were identified using the Louvain clustering algorithm (resolution = 0.5) on a shared nearest neighbor graph. Expression of known tumor markers (FOXI1 and LINC01187 for chRCC and oncocytoma,^8^ TRIM63 for TRCC,^6^ ITGB8 for pRCC,^154^ PAX8 for ESCRCC,^155^ PECAM1 and ENG for endothelial cells, ACTA2 and RGS5 for SMC, PTPRC for immune cells, FABP4 and PLN1 for adipocytes) were used to annotate cell clusters. Since AMLs consist of blood vessels, smooth muscle cells, and adipocytes, cells expressing markers of SMC, endothelial cells and adipocytes were considered “Tumor” for AML libraries. Annotations of non-tumor cells were complemented by prediction using a published snRNA-seq dataset of human normal kidney^35^ as reference. Re-clustering of all tumor cells with resolution = 0.2 was done for each sample to identify tumor subclusters. Cell cycle phase was assigned based on scoring of expression of G2/M and S phase markers.^156^

To visualize clustering patterns of cell types from all RCC subtypes, a subset of 2000 cells of each library were randomly selected and pooled together. Downstream analyses from normalization to dimension reduction (UMAP) followed the same procedure as for individual libraries. To show similarity and dissimilarity among tumor cells of different subtypes, tumor cells of all libraries except AMLs were pooled and PCA was performed on top 500 highly variable genes.

#### Cell-of-origin prediction and bulk data projection

Putative cell-of-origin of each profiled RCC subtype was inferred following previously published procedure.^36^ A random forest model was trained with a snRNA-seq dataset of normal kidney epithelial cells (only HVGs were used; 300 cells were randomly selected for overrepresented clusters to minimize bias due to unbalanced sample sizes). The model was then applied to snRNA-seq data of RCC tumor cells to predict their closest normal cell types (putative COO). In addition, prediction based on bulk RNA-seq data of ccRCC and rare RCCs of this study was performed by first applying rank-based inverse normal transformation. Random forest classifier was then built on transformed sn data of normal kidney epithelial cells; transformed bulk data of RCCs were then used to predict putative COO.

#### Proteogenomic signature of RCC subtypes

To identify subtype-specific markers, differentially expression (DE) analysis was performed for each of the rare RCC subtypes profiled by RNA-seq and proteomics. For RNA-seq, the input data is voom-transformed data with associated precision weights.^156^ For proteomic data, the input was normalized and log2 transformed data. Input data was fit into linear models with limma,^157^ and contrasts were constructed to compare each subtype with the average of all other subtypes. Top 100 upregulated genes ranked by *p* value were selected for each subtype (for RNA, additional filter of logFC>2, adjusted *p* value < 0.01 were applied) as the signature gene set. To visualize expression of the gene sets as “metagenes”, *Z* score was calculated for each gene of each gene set and the average was used to make the heatmap.

#### Gene set/Pathway enrichment analysis

To visualize the pathway enriched across different RCC subtypes, differential expression analysis was first performed to identify differential expressed RNAs and proteins, respectively. Then, gene set enrichment analysis was performed to identify enriched concepts. GSEA for RNA and proteomics data were performed through the ClusterProfiler R package.^158^ The annotation of concepts are fetched from REATCOME,^159^ MSigDB^160^ and KEGG.^161^

#### Phosphorylation site level enrichment analysis

Based on the results of differential expression analysis with phosphorylation sites intensity data between each tumor subtype and normal samples, we performed phosphosite-specific signature enrichment analysis^114^ (PTM-SEA) to identify dysregulated phosphorylation-driven pathways. To adequately account for both magnitude and variance of measured phosphosite abundance, we use *t.mod,* moderated *t statistic* resulting from the ebayes function as ranking for PTM-SEA. We queried the PTM signature database (PTMsigDB) v1.9.0 downloaded from http://prot-shiny-vm.broadinstitute.org:3838/ptmsigdb-app/using the Uniprot ID plus residue location as identifier. We call the functions of PTM-SEA available on GitHub (https://github.com/broadinstitute/ssGSEA2.0) within R. The following parameters were used to run PTM-SEA; weight: 1, statistic: ‘‘area.under.RES’’, output.score.type: ‘‘NES’’, nperm: 1000, min.overlap: 5, correl.type: ‘‘z.score’’

The sign of the normalized enrichment score (NES) calculated for each signature corresponds to the sign of the tumor-normal log fold change. *p*-values for each signature were derived from 1,000 random permutations and further adjusted for multiple hypothesis testing using the method proposed by Benjamini & and Hochberg (Benjamini and Hochberg, 1995). Due to limited sample size, signatures with a lenient FDR threshold 0.2 were considered to be differential. Pathway signatures that are significant in at least one of the 9 comparisons are displayed in the bubble plot (Figure 3C). Kinase signatures are plotted in pseudo volcano plots in high vs. low wGII non-ccRCC tumor comparison (Figure 3D) and chromosome 7 gain vs. no gain non-ccRCC (pRCC, TRCC and ESCRCC) comparison (Figure S6g).

#### Metabolic pathway enrichment analysis with metabolites and enzymes

Upregulated and downregulated metabolites and proteins (q value < 0.05, absolute value of log2 fold change >1) in ccRCC, pRCC type-1, AML, RO type2 combined with RO variants compared to normal samples are sent to IMPaLA^116^ (http://impala.molgen.mpg.de/) for pathway over-representation analysis separately. KEGG ID is the metabolite identifier and gene symbol is the protein identifier. We focused on HumanCyc metabolic pathways^162^ (https://humancyc.org/) from the analysis results (Figure 4C).

#### Transcription factor regulon identification

pySCENIC (https://pyscenic.readthedocs.io/en/latest/) and R package SCENIC (version 1.1.2) were used for transcription factor regulon identification. Input of this analysis are our raw bulk RNA-Seq data and the raw proteome data. We used an in-house constructed pipeline via combining GRNBoost2 from pySCENIC algorithms and RSCENIC algorithms with default parameters. To predict the regulons, we used human v9 motif collection, as well as both hg38__refseq-r80__10kb_up_and_down_tss.mc9nr.feather and hg38__refseq-r80__500bp_u-p_and_100bp_down_tss.mc9nr.feather databases from the cisTarget (https://resources.aertslab.org/cistarget/databases/homo_sapiens/hg38/refseq_r80/mc9nr/gene_based/). The resulting AUC scores matrix was used for downstream analysis(Heatmap and RSS plot)

#### Kinase-substrate co-regulation analysis

Kinase and residue level substrate relationships were collected from OmniPath^99^ (OmniPath:: Intra- & intercellular signaling knowledge (omnipathdb.org)) using R package *OmnipathR*^121^ for comprehensive coverage. Keeping only “phosphorylation” modifications, we get 40,122 kinase-substrate pairs. After mapping the proteome data to kinases and the phosphoproteome data to substrates (with site level resolution), we gathered 9,932 kinase-substrate pairs for joint differential analysis. We derive two-dimensional *Z* score vectors for both kinase ( ) and substrate ( ):

Using lmFit and eBayes function from the limma package, we regress kinase protein abundance/phosphorylation site intensity data separately against sample grouping (low wGII or high wGII) with tumor purity adjusted as a covariate. The resultant statistic is assigned as the *Z* score for kinase and substrate respectively. We model the distribution of all Z scores derived from all the kinase-substrate pairs as a mixture of pairs which are up- or down-regulated between conditions (e.g., high or low wGII) and pairs which are non-differentially regulated. Assuming the Z scores of the differentially regulated proteins follow an empirical distribution and the Z scores of the non-differentially regulated proteins follow an empirical null distribution, we can write the observed distribution for *Z* score as:

$$f\left(Z\right)=p_{0}f_{0}\left(Z\right)+p_{1}f_{1}\left(Z\right)$$

where are the proportion of the non-differentially and differentially regulated pairs in the data. We have $p_{0}+p_{1}=1$. To estimate the distribution of the non-differentially regulated pairs, we simulate the null distribution of Z scores by randomly permuting the measurements of the two conditions for 200 times. Following this mixture deconvolution, we calculate the posterior probability of being differentially regulated for each protein with the following equation:

$$p_{1}\left(Z\right)=1-p_{0}f_{0}\left(Z\right)/f\left(Z\right)$$

is estimated by taking the ratio of densities at:

$$Z_{00}=<Z_{k}=0,{\;Z}_{s}=0>$$

$$p_{0\;}=f\left(Z_{00}\right)/f_{0}\left(Z_{00}\right)$$

The local false discovery rate (fdr) is computed as:

$$fdr\left(Z\right)=p_{0}f_{0}\left(Z\right)/f\left(Z\right)$$

Throughout this modeling, we only used the proteins and phosphosites with missing data in fewer than 6 samples for construction of reliable *Z* score. The R implementation of KSA2D^115^ can be found in https://www.github.com/ginnyintifa/KSA2D.

pRCC MTSCC specific marker validation with external data (Figures 7C and 7D)

Protein expression of a pRCC and MTSCC cohort was fetched from the previous publication.^23^ Differential expression analysis was performed in the same way as described in the Differential Expression Analysis section. One outliner MTSCC sample was removed due to absence of canonical copy number loss events of chr1 chr6, and chr9.

#### Survival analysis

The R package survival^122^ was used to perform survival analysis. The Kaplan-Meier curve of overall survival was used to compare the prognosis among subtypes (function survfit). The standard multivariate Cox-proportional hazard modeling (function coxph) was applied to calculate hazard ratio between categories of interest (e.g., high or low expression of certain gene, methylation groups, wGII groups). Age, gender, race, tumor stage and tumor purity were adjusted as the model covariates. Log rank test was used to test the differential survival outcomes between categorical variables.

## Supplemental information

Document S1. Figures S1–S7

Table S1. Sample annotation table, related to Figure 1 Clinical characteristics of patients, data availability of samples, WGS, WES, and RNA-seq depth.

Table S2. RNA protein tables, related to Figure 1 Processed RNA and protein tables used for downstream analysis.

Table S3. Sample molecular features, related to Figure 1 Sample molecular characteristics including somatic mutations, copy number variation, methylation probes matrix, immune deconvolution results, differential expression analysis, and gene set enrichment analysis results.

Table S4. High-wGII tumor markers, related to Figure 1 Differential expression analysis and gene set enrichment analysis (GSEA) results comparing high-wGII and low-wGII groups in RNA and protein data.

Table S5. Single-nuclei RNA-seq, related to Figure 2 Sample information for snRNA-seq data and cell clustering results.

Table S6. Phosphoproteomics analysis, related to Figure 3 Protein phosphorylation analysis including site level differential expression analysis, PTM-SEA, kinase-substrate co-regulation analysis, and CLUMPS-PTM analysis results.

Table S7. Glycoproteomics analysis, related to Figure 4 Protein glycosylation analysis including differential expression analysis and cell type enrichment analysis.

Table S8. Metabolomics analysis, related to Figure 5 Differential expression and metabolite-enzyme joint enrichment analysis results.

Table S9. Papillary RCC characterization, related to Figure 6 Differential expression analysis of pRCC using RNA, protein, and phosphorylation including external cohort comparison.

Table S10. Oncocytic tumor characterization, related to Figure 7 Differential expression and transcription regulon analysis of oncocytic tumors using RNA and protein data.

Document S2. Article plus supplemental information
