# Supplementary material for: Comprehensive proteogenomic characterization of rare kidney tumors
Source: Cell Rep Med. 2024 May 3;5(5):101547. doi: 10.1016/j.xcrm.2024.101547 (PMC11148773; doi:10.1016/j.xcrm.2024.101547)
Supplement: Document S1. Figures S1–S7 [file mmc1.pdf]

Cell Reports Medicine, Volume *5*

Supplemental information

Comprehensive proteogenomic characterization of rare kidney tumors

Ginny Xiaohe Li, Lijun Chen, Yi Hsiao, Rahul Mannan, Yuping Zhang, Jie Luo, Francesca Petralia, Hanbyul Cho, Noshad Hosseini, Felipe da Veiga Leprevost, Anna Calinawan, Yize Li, Shankara Anand, Aniket Dagar, Yifat Geffen, Chandan Kumar-Sinha, Seema Chugh, Anne Le, Sean Ponce, Shenghao Guo, Cissy Zhang, Michael Schnaubelt, Nataly Naser Al Deen, Feng Chen, Wagma Caravan, Andrew Houston, Alex Hopkins, Chelsea J. Newton, Xiaoming Wang, Daniel A. Polasky, Sarah Haynes, Fengchao Yu, Xiaojun Jing, Siqi Chen, Ana I. Robles, Mehdi Mesri, Mathangi Thiagarajan, Eunkyung An, Gad A. Getz, W. Marston Linehan, Galen Hostetter, Scott D. Jewell, Daniel W. Chan, Pei Wang, Gilbert S. Omenn, Rohit Mehra, Christopher J. Ricketts, Li Ding, Arul M. Chinnaiyan, Marcin P. Cieslik, Saravana M. Dhanasekaran, Hui Zhang, Alexey I. Nesvizhskii, and Clinical Proteomic Tumor Analysis Consortium

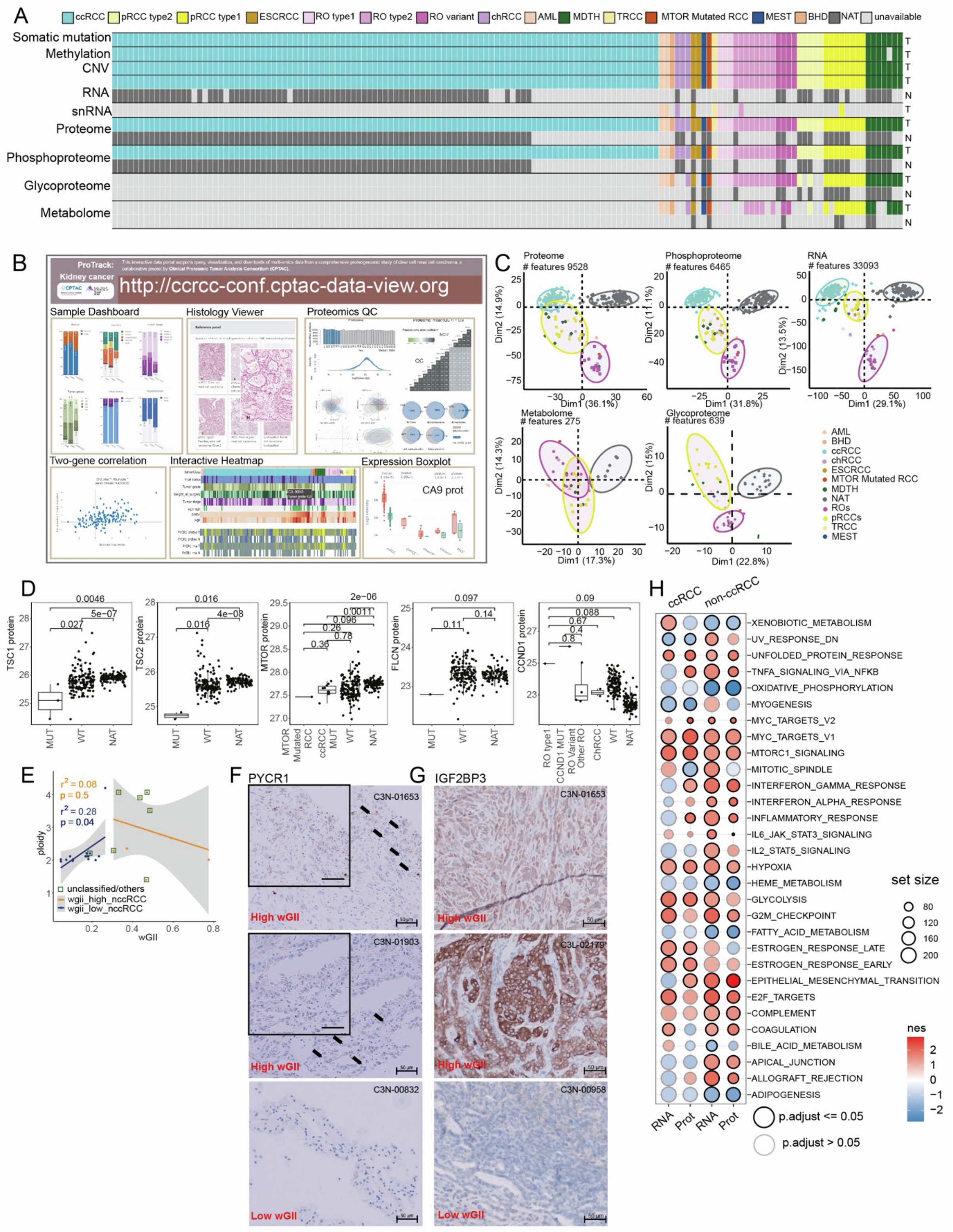
A
ccRCC pRCC type2 pRCC type1 ESCRCC RO type1 RO type2 RO variant chRCC AML MDTH TRCC MTOR Mutated RCC MEST BHD NAT unavailable
Somatic mutation
Methylation
CNV
RNA
snRNA
Proteome
Phosphoproteome
Glycoproteome
Metabolome
B
ProTrack: Kidney cancer
http://ccrcc-conf.cptac-data-view.org
Sample Dashboard
Histology Viewer
Proteomics QC
Two-gene correlation
Interactive Heatmap
Expression Boxplot
CA9 prot
C
Proteome # features 9528
Phosphoproteome # features 6465
RNA # features 33093
Metabolome # features 275
Glycoproteome # features 639
Dim1 (36.1%)
Dim2 (14.9%)
Dim1 (31.8%)
Dim2 (11.1%)
Dim1 (29.1%)
Dim2 (13.5%)
Dim1 (17.3%)
Dim2 (14.3%)
Dim1 (22.8%)
Dim2 (15%)
AML
BHD
ccRCC
chRCC
ESCRCC
MTOR Mutated RCC
MDTH
NAT
ROs
pRCCs
TRCC
MEST
D
TSC1 protein
TSC2 protein
MTOR protein
FLCN protein
CCND1 protein
MUT
WT
NAT
MTOR Mutated RCC
ccRCC
RO type1
CCND1 MUT
RO Variant
Other RO
ChRCC
E
r² = 0.08
p = 0.5
r² = 0.28
p = 0.04
ploidy
wGII
unclassified/others
wgii_high_nccRCC
wgii_low_nccRCC
F
PYCR1
C3N-01653
C3N-01903
C3N-00832
High wGII
Low wGII
G
IGF2BP3
C3N-01653
C3L-02179
C3N-00958
High wGII
Low wGII
H
ccRCC
non-ccRCC
XENOBIOTIC_METABOLISM
UV_RESPONSE_DN
UNFOLDED_PROTEIN_RESPONSE
TNFA_SIGNALING_VIA_NFKB
OXIDATIVE_PHOSPHORYLATION
MYOGENESIS
MYC_TARGETS_V2
MYC_TARGETS_V1
MTORC1_SIGNALING
MITOTIC_SPINDLE
INTERFERON_GAMMA_RESPONSE
INTERFERON_ALPHA_RESPONSE
INFLAMMATORY_RESPONSE
IL6_JAK_STAT3_SIGNALING
IL2_STAT5_SIGNALING
HYPOXIA
HEME_METABOLISM
GLYCOLYSIS
G2M_CHECKPOINT
FATTY_ACID_METABOLISM
ESTROGEN_RESPONSE_LATE
ESTROGEN_RESPONSE_EARLY
EPITHELIAL_MESENCHYMAL_TRANSITION
E2F_TARGETS
COMPLEMENT
COAGULATION
BILE_ACID_METABOLISM
APICAL_JUNCTION
ALLOGRAFT_REJECTION
ADIPOGENESIS
RNA Prot RNA Prot
set size
80
120
160
200
nes
p.adjust <= 0.05
p.adjust > 0.05

Figure S1. Characterization of renal cell carcinoma proteogenomic aberration landscape reveals association between survival and copy number-based genome instability, and wGII biomarkers validation, Related to Figure 1

A. Sample availability of bulk experiments included in this study.

B. ProTrack modules include Sample Dashboard, Histology Viewer, Proteomic QC, Two-gene Correlation, Interactive Heatmap and Expression Boxplots.

C. PCA plots show distribution of samples with regard to PC1 (x axis) and PC2 (y axis) in global proteome, phosphoproteome, RNA, metabolome and glyco-enriched glycoproteome, stratified by tumor/normal condition and ccRCC/non-ccRCC cohorts, colored by TMT plexes. PTM data are represented by the site-level aggregation.

D. Boxplots show protein expression between mutated and wild type groups for kinases TSC1, TSC2, MTOR, FLCN and CCND1. P-values were calculated using Wilcoxon rank sum test.

E. Scatter plot shows ploidy plotted against wGII for non-ccRCC samples. P-values were calculated for Pearson correlation coefficient.

F. PYCR1 RNA-ISH staining in 2 high wGII non-ccRCC cases and 1 low wGII non-ccRCC case.

G. IGF2BP3 IHC staining in 2 high wGII non-ccRCC cases and 1 low wGII non-ccRCC case.

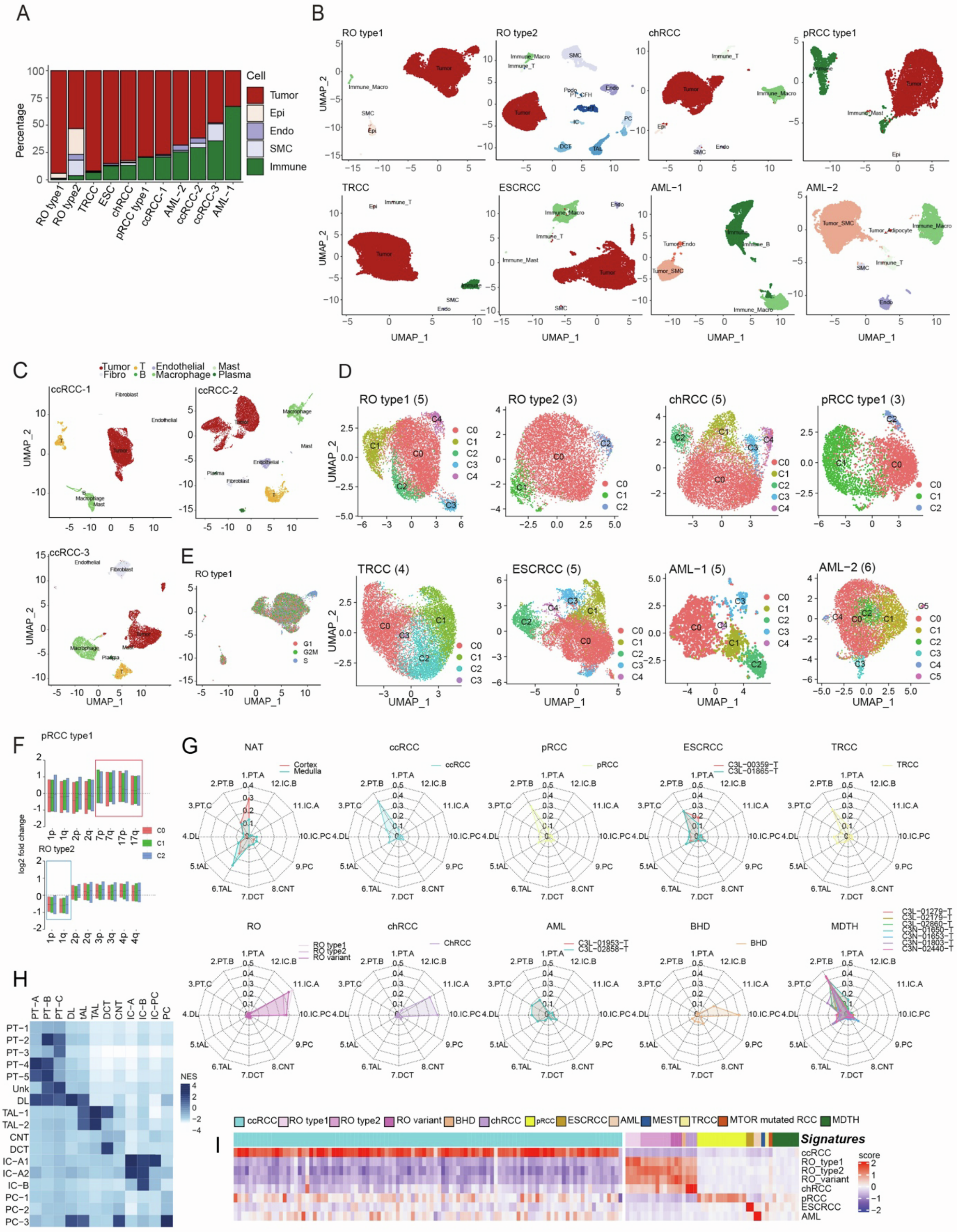

A
B
C
D
E
F
G
H
I
Cell
Tumor
Epi
Endo
SMC
Immune
Percentage
RO type1
RO type2
TRCC
ESC
chRCC
pRCC type1
ccRCC-1
AML-2
ccRCC-2
ccRCC-3
AML-1
pRCC type1
ccRCC-1
ccRCC-2
ccRCC-3
RO type1 (5)
RO type2 (3)
chRCC (5)
pRCC type1 (3)
TRCC (4)
ESCRCC (5)
AML−1 (5)
AML−2 (6)
UMAP_1
UMAP_2
log2 fold change
NAT
ccRCC
pRCC
ESCRCC
TRCC
RO
chRCC
AML
BHD
MDTH
NES
Signatures
ccRCC
RO_type1
RO_type2
RO_variant
chRCC
pRCC
ESCRCC
AML
score

Figure S2. Delineation of tumor transcriptomic heterogeneity, immune infiltration status and tumor cell of origin by single nuclei RNAsequencing, Related to Figure 2

A. Stacked bar plot shows the cell type composition of the different samples. Non-ccRCC tumors (except AML) showed higher tumor purity and lower immune infiltration, while in comparison 3 ccRCC samples (C3L−00606−01, C3N−00148−03, C3N−00149−02; described in the companion ccRCC study) and the 2 AML showed higher levels of immune infiltration.

B. Two-dimensional UMAP visualization of snRNA-seq data for each of the 8 tumors, colored by clusters.

C. Two-dimensional UMAP visualization of snRNA-seq data for 3 ccRCC samples from the discovery cohort, colored by cell types.

D. Two-dimensional UMAP visualization of snRNA-seq data for each of the 8 tumors, colored by tumor subclusters.

E. Two-dimensional UMAP visualization of snRNA-seq data for the RO type-1 tumor, colored by cell cycle classification predicted based on expression of phase specific markers.

F. Box plots display fold change (log2 scale) of snRNA-seq expression of genes located in selected chromosomal arms between tumor subcluster and other cells of the pRCC type-1 sample (top) and the RO type2 sample (bottom)

G. Radar plot shows the probability of cell-of-origin predicted by random forest classifier for different tumor subclusters for each of the RCC subtypes using bulk RNAseq data.

H. Heatmap shows overlaps of renal epithelial cell types identified in Lake et al. (snRNA-seq) and Zhang et al. (scRNA-seq). Color scale indicates enrichment of cell markers identified from Zhang et al dataset in Lake et al. data set.

I. Heatmap shows scores of subtype-specific gene signatures derived from top 50 most upregulated transcripts for each tumor subtype.

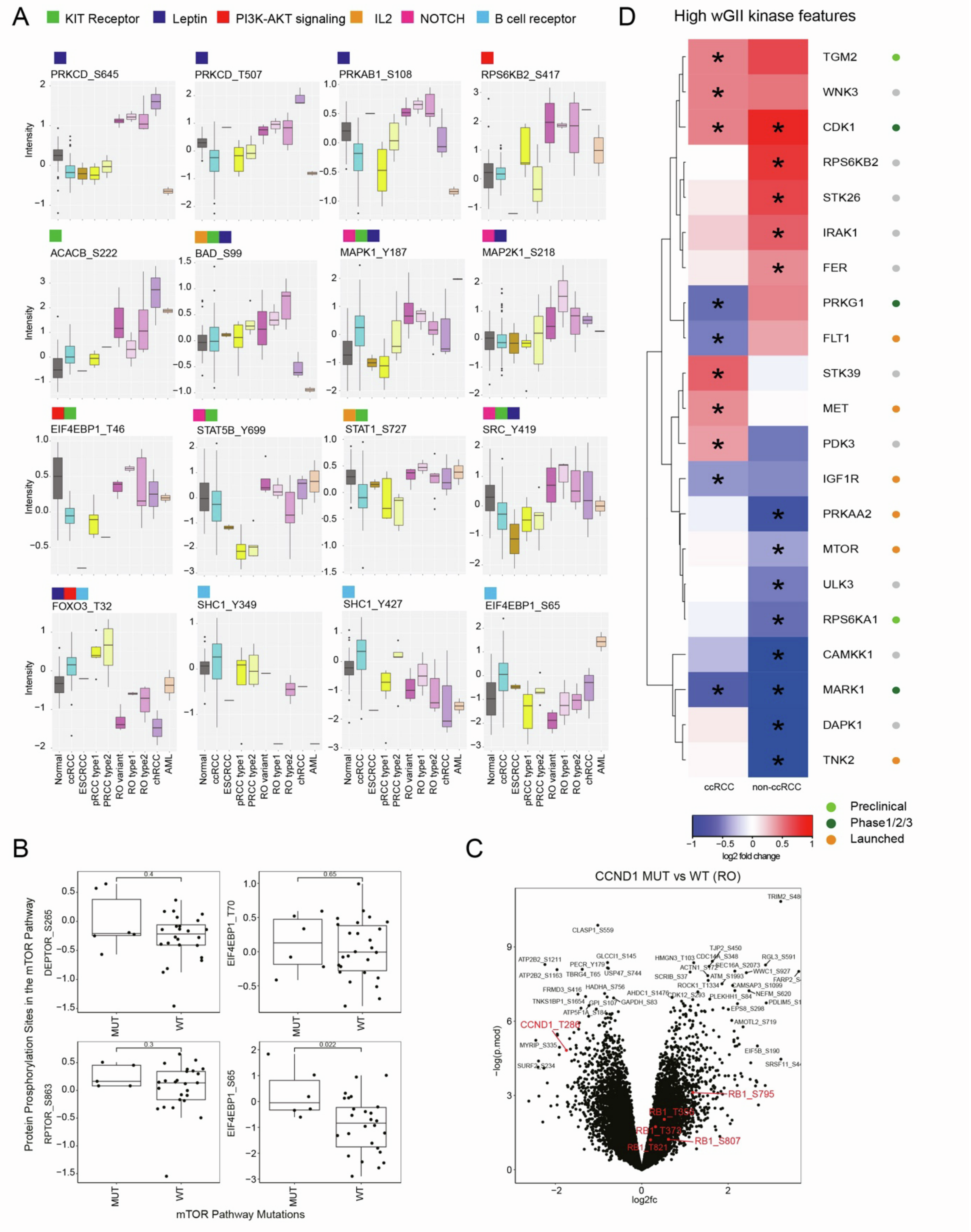

A
KIT Receptor
Leptin
PI3K-AKT signaling
IL2
NOTCH
B cell receptor
PRKCD_S645
PRKCD_T507
PRKAB1_S108
RPS6KB2_S417
ACACB_S222
BAD_S99
MAPK1_Y187
MAP2K1_S218
EIF4EBP1_T46
STAT5B_Y699
STAT1_S727
SRC_Y419
FOXO3_T32
SHC1_Y349
SHC1_Y427
EIF4EBP1_S65
Intensity
Normal
ccRCC
ESCRCC
pRCC type1
PRCC type2
RO variant
RO type1
RO type2
chRCC
AML
D
High wGII kinase features
TGM2
WNK3
CDK1
RPS6KB2
STK26
IRAK1
FER
PRKG1
FLT1
STK39
MET
PDK3
IGF1R
PRKAA2
MTOR
ULK3
RPS6KA1
CAMKK1
MARK1
DAPK1
TNK2
ccRCC
non-ccRCC
Preclinical
Phase1/2/3
Launched
log2 fold change
B
Protein Prosphorylation Sites in the mTOR Pathway
DEPTOR_S265
EIF4EBP1_T70
RPTOR_S863
EIF4EBP1_S65
MUT
WT
mTOR Pathway Mutations
C
CCND1 MUT vs WT (RO)
CCND1_T286
RB1_S795
RB1_S807
RB1_T373
RB1_T821
−log(p.mod)
log2fc

Figure S3. Phosphoproteomic changes in non ccRCC tumors, Related to Figure 3

A. Boxplots show phosphorylation intensity of the indicated phosphosites stratified by tumor subtypes. Color box on the top indicates signaling pathways the phosphorylation belongs to.

B. Boxplots show different levels of phosphosites of intermediates in mTOR pathway including DEPTOR_S265, RPTOR_S863, EIF4EBP1_T70 and EIF4EBP1_S85 between different mTOR pathway genes mutation status (TSC1, TSC2, FLCN, or mTOR mutations). P-values were calculated using Wilcoxon rank sum test.

C. Volcano plot shows differentially regulated phosphorylation sites between CCND1 mutated and wild type RO samples. Known CCND1 mutation related sites are highlighted in red. RB1 phosphorylation is downstream of CCND1.

D. Heatmap shows the log2 fold change of kinases between samples with high and low wGII in ccRCC and non-ccRCC. Significantly differentially expressed kinases (p-values less than 0.05 using Limma) are labeled. Color of the dots represents the drug discovery stage of kinases.

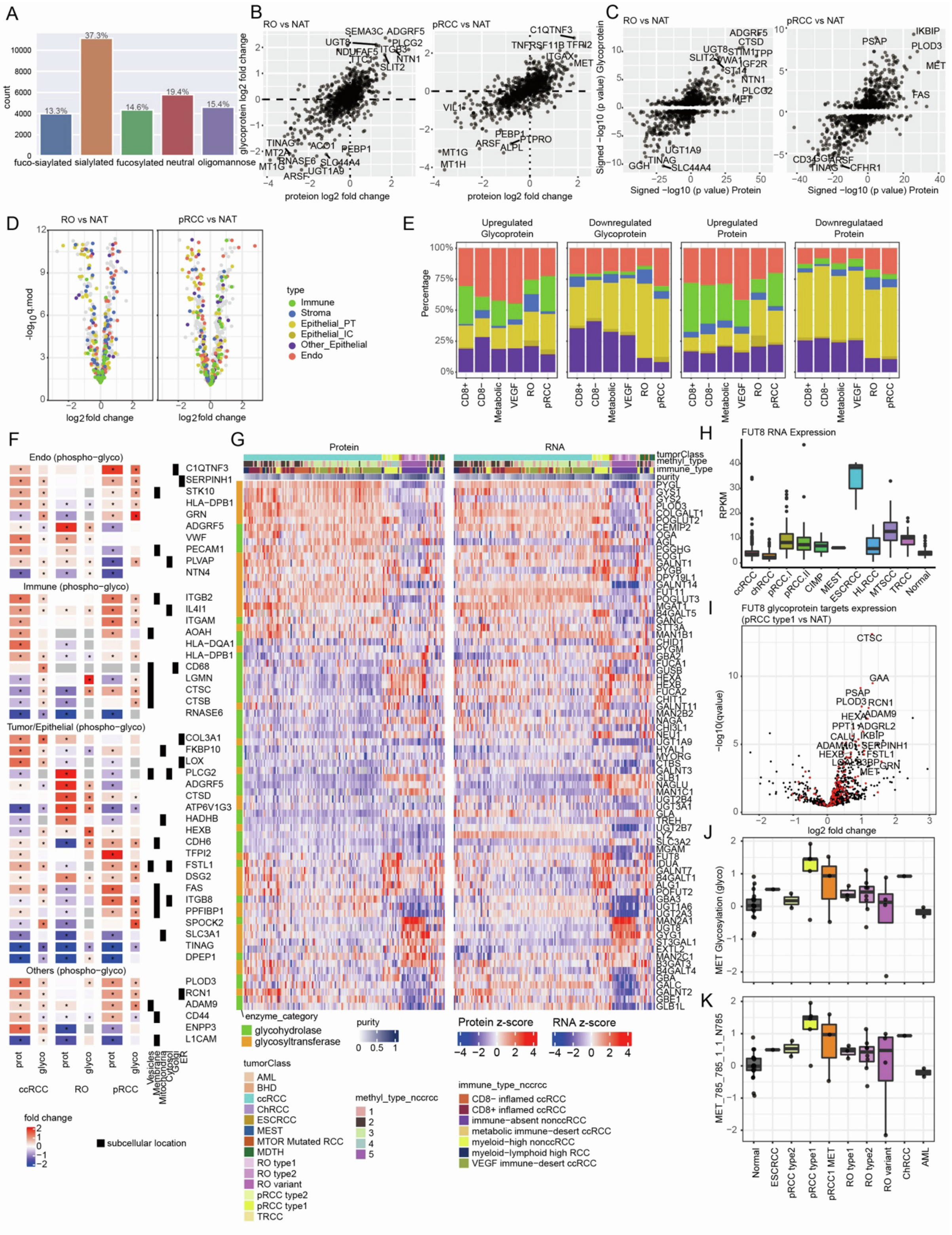
A
count
fuco-siaylated sialylated fucosylated neutral oligomannose
13.3% 37.3% 14.6% 19.4% 15.4%
B
RO vs NAT
pRCC vs NAT
glycoprotein log2 fold change
proteion log2 fold change
C
RO vs NAT
pRCC vs NAT
Signed −log10 (p value) Glycoprotein
Signed −log10 (p value) Protein
D
RO vs NAT
pRCC vs NAT
log2 fold change
type
Immune
Stroma
Epithelial_PT
Epithelial_IC
Other_Epithelial
Endo
E
Upregulated Glycoprotein
Downregulated Glycoprotein
Upregulated Protein
Downregulataed Protein
Percentage
CD8+ CD8− Metabolic VEGF RO pRCC
F
Endo (phospho−glyco)
Immune (phospho−glyco)
Tumor/Epithelial (phospho−glyco)
Others (phospho−glyco)
prot glyco prot glyco prot glyco
ccRCC RO pRCC
Vesicles Membrane Mitochondria Cytosol Golgi ER
fold change
subcellular location
G
Protein
RNA
tumorClass
methyl_type
immune_type
purity
enzyme_category
glycohydrolase
glycosyltransferase
Protein z-score
RNA z-score
AML BHD ccRCC ChRCC ESCRCC MEST MTOR Mutated RCC MDTH RO type1 RO type2 RO variant pRCC type2 pRCC type1 TRCC
methyl_type_nccrcc
immune_type_nccrcc
CD8− inflamed ccRCC
CD8+ inflamed ccRCC
immune−absent nonccRCC
metabolic immune−desert ccRCC
myeloid−high nonccRCC
myeloid−lymphoid high RCC
VEGF immune−desert ccRCC
H
FUT8 RNA Expression
RPKM
ccRCC chRCC pRCC.I pRCC.II CIMP MEST ESCRCC HLRCC MTSCC TRCC Normal
I
FUT8 glycoprotein targets expression (pRCC type1 vs NAT)
−log10(qvalue)
log2 fold change
J
MET Glycosylation (glyco)
K
MET_785_785_1_1_N785
Normal ESCRCC pRCC type2 pRCC type1 pRCC1 MET RO type1 RO type2 RO variant ChRCC AML

Figure S4. RCC glycoproteome reflects tumor immune infiltration and angiogenesis, Related to Figure 4

A. Bar plot shows distribution of glycoforms found in phospho-enriched samples.

B. Scatter plots show glycosylation change versus its corresponding protein (fold change, glyco). Right:RO. Left: pRCC.

C. Glycosylation changes versus its corresponding protein (signed p-value, glyco). Right: RO. Left: pRCC.

D. Volcano plots show differential expressed glycoproteins in RO (left) and pRCC (right). Colored by cell-type specific expression from previous single cell RNAseq data.

E. Stacked bar plots summarize differentially expressed cell type specific glycoproteins (left) and proteins (right) in phospho-enriched samples.

F. Heatmap of differentially expressed cell type specific glycoprotein markers in phospho-enriched samples. * denotes the marker expression difference is significant (adjusted q-value < 0.05 using Limma).

G. Protein (left) and RNA (right) expression of glycosylation enzymes in kidney tumors. The expression is converted into z-score across all samples.

H. FUT8 RNA expression across kidney cancers in combined TCGA and in house MCTP cohort.

I. Volcano plot for glycosylation changes of putative FUT8 targets in pRCC type-1. Red dots are putative FUT8 target proteins collected from previous studies.

J. MET glycosylation in glyco-enriched samples.

K. MET_N785 glycosylation in glyco-enriched samples

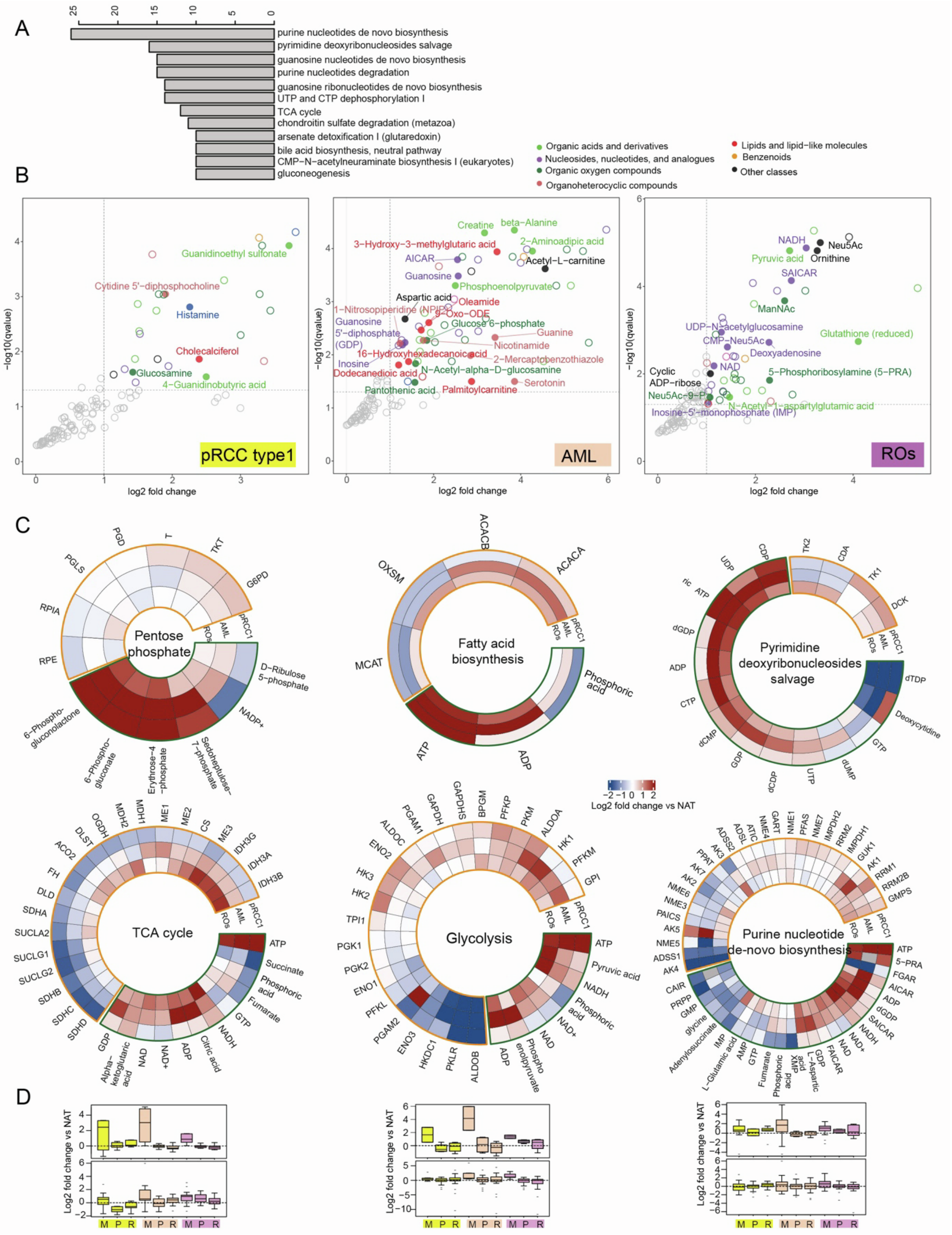
A
purine nucleotides de novo biosynthesis
pyrimidine deoxyribonucleosides salvage
guanosine nucleotides de novo biosynthesis
purine nucleotides degradation
guanosine ribonucleotides de novo biosynthesis
UTP and CTP dephosphorylation I
TCA cycle
chondroitin sulfate degradation (metazoa)
arsenate detoxification I (glutaredoxin)
bile acid biosynthesis, neutral pathway
CMP−N−acetylneuraminate biosynthesis I (eukaryotes)
gluconeogenesis
B
Organic acids and derivatives
Nucleosides, nucleotides, and analogues
Organic oxygen compounds
Organoheterocyclic compounds
Lipids and lipid−like molecules
Benzenoids
Other classes
−log10(qvalue)
log2 fold change
Guanidinoethyl sulfonate
Cytidine 5'−diphosphocholine
Histamine
Cholecalciferol
Glucosamine
4−Guanidinobutyric acid
pRCC type1
Creatine
beta−Alanine
2−Aminoadipic acid
3−Hydroxy−3−methylglutaric acid
AICAR
Acetyl−L−carnitine
Guanosine
Phosphoenolpyruvate
Aspartic acid
Oleamide
1−Nitrosopiperidine (NPIP)
9−Oxo−ODE
Guanosine 5'−diphosphate (GDP)
Glucose 6−phosphate
Guanine
Nicotinamide
16−Hydroxyhexadecanoic acid
2−Mercaptobenzothiazole
Inosine
Dodecanedioic acid
N−Acetyl−alpha−D−glucosamine
Serotonin
Pantothenic acid
Palmitoylcarnitine
AML
NADH
Neu5Ac
Pyruvic acid
Ornithine
SAICAR
ManNAc
UDP−N−acetylglucosamine
CMP−Neu5Ac
Glutathione (reduced)
Deoxyadenosine
NAD
Cyclic ADP−ribose
5−Phosphoribosylamine (5−PRA)
Neu5Ac−9−P
N−Acetyl−L−aspartylglutamic acid
Inosine−5'−monophosphate (IMP)
ROs
C
Pentose phosphate
Fatty acid biosynthesis
Pyrimidine deoxyribonucleosides salvage
TCA cycle
Glycolysis
Purine nucleotide de-novo biosynthesis
Log2 fold change vs NAT
D
Log2 fold change vs NAT
M P R M P R M P R

Figure S5. Metabolomic aberrations across RCC subtypes, Related to Figure 5

A. Barplot shows the top 10 metabolic pathways with the most number of compounds identified.

B. Volcano plots show upregulated compounds in pRCC type-1 (left), AML (middle) and ROs (right), with compounds of different categories colored. Only uniquely DE compounds are labeled and marked with solid circles.

C. Metabolograms depicting select metabolic pathways to show the coordinated regulation of compounds (metabolites abundance; within green border) and the corresponding enzymes (proteins abundance; within orange border) in pRCC type-1 (outer circle), AML (middle circle) and ROs (inner circle). Color represents abundance fold change in log2 scale between respective tumor class and NATs.

D. Boxplots compare fold changes of metabolites (M), proteins (P) and mRNAs (R) across tumor subtypes within each pathway. Ordered the same as panel C.

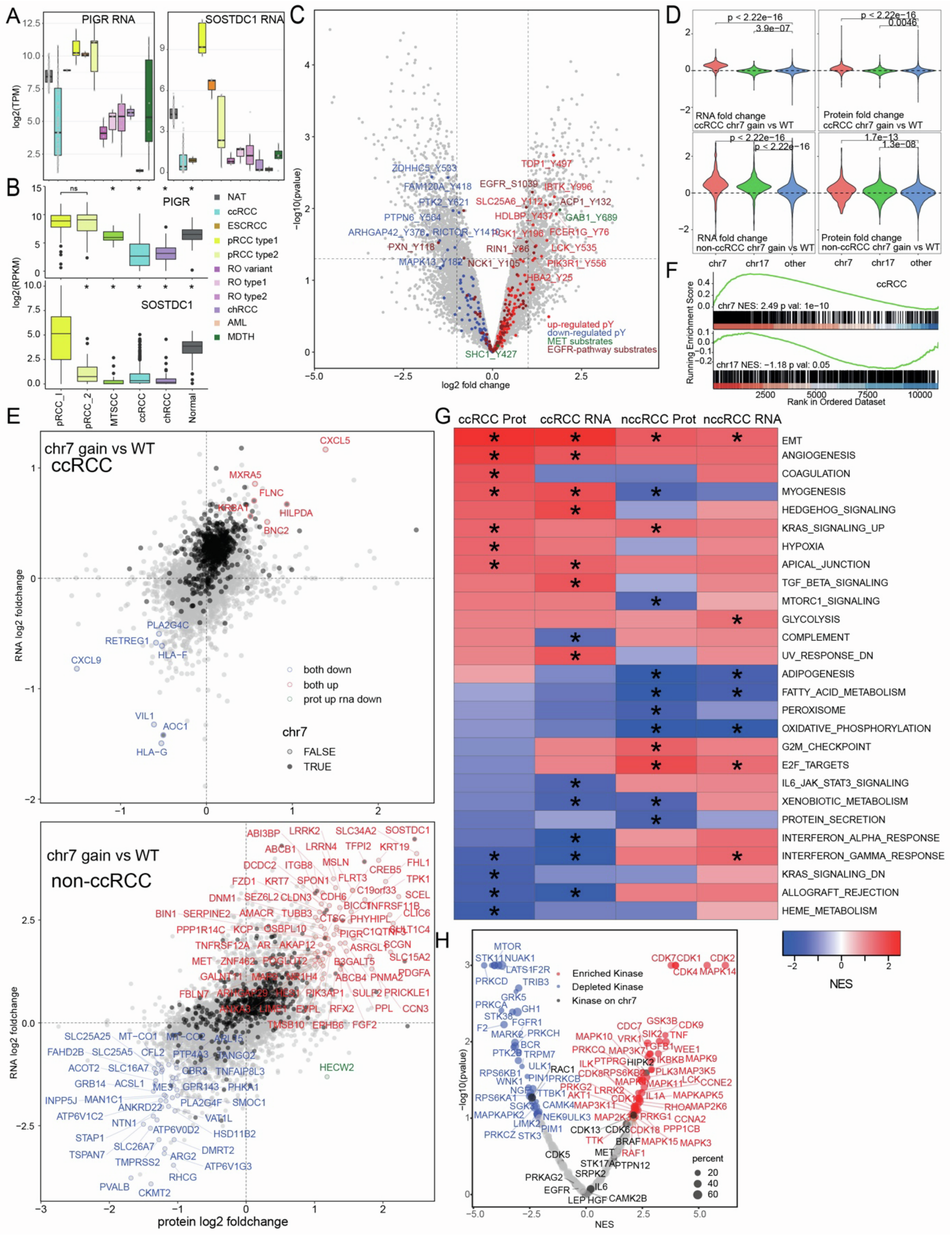
A
PIGR RNA
SOSTDC1 RNA
log2(TPM)
B
PIGR
SOSTDC1
log2(RPKM)
ns
NAT
ccRCC
ESCRCC
pRCC type1
pRCC type2
RO variant
RO type1
RO type2
chRCC
AML
MDTH
pRCC_I
pRCC_2
MTSCC
ccRCC
chRCC
Normal
C
−log10(pvalue)
log2 fold change
ZDHHC5_Y533
FAM120A_Y418
PTK2_Y621
PTPN6_Y564
ARHGAP42_Y376
RICTOR_Y1410
PXN_Y118
MAPK13_Y182
NCK1_Y105
SHC1_Y427
TDP1_Y497
EGFR_S1039
BTK_Y996
SLC25A6_Y112
ACP1_Y132
HDLBP_Y437
GAB1_Y689
PGK1_Y196
FCER1G_Y76
RIN1_Y86
LCK_Y535
PIK3R1_Y556
HBA2_Y25
up-regulated pY
down-regulated pY
MET substrates
EGFR-pathway substrates
D
p < 2.22e−16
3.9e−07
0.0046
RNA fold change ccRCC chr7 gain vs WT
Protein fold change ccRCC chr7 gain vs WT
p < 2.22e−16
1.7e−13
1.3e−08
RNA fold change non-ccRCC chr7 gain vs WT
Protein fold change non-ccRCC chr7 gain vs WT
chr7
chr17
other
F
ccRCC
Running Enrichment Score
chr7 NES: 2.49 p val: 1e−10
chr17 NES: −1.18 p val: 0.05
Rank in Ordered Dataset
E
chr7 gain vs WT
ccRCC
RNA log2 foldchange
CXCL5
MXRA5
FLNC
KRBA1
HILPDA
BNC2
PLA2G4C
RETREG1
HLA-F
CXCL9
VIL1
AOC1
HLA-G
both down
both up
prot up rna down
chr7
FALSE
TRUE
chr7 gain vs WT
non-ccRCC
protein log2 foldchange
HECW2
G
ccRCC Prot
ccRCC RNA
nccRCC Prot
nccRCC RNA
EMT
ANGIOGENESIS
COAGULATION
MYOGENESIS
HEDGEHOG_SIGNALING
KRAS_SIGNALING_UP
HYPOXIA
APICAL_JUNCTION
TGF_BETA_SIGNALING
MTORC1_SIGNALING
GLYCOLYSIS
COMPLEMENT
UV_RESPONSE_DN
ADIPOGENESIS
FATTY_ACID_METABOLISM
PEROXISOME
OXIDATIVE_PHOSPHORYLATION
G2M_CHECKPOINT
E2F_TARGETS
IL6_JAK_STAT3_SIGNALING
XENOBIOTIC_METABOLISM
PROTEIN_SECRETION
INTERFERON_ALPHA_RESPONSE
INTERFERON_GAMMA_RESPONSE
KRAS_SIGNALING_DN
ALLOGRAFT_REJECTION
HEME_METABOLISM
NES
H
Enriched Kinase
Depleted Kinase
Kinase on chr7
percent
−log10(pvalue)
NES
MTOR
CDK7
CDK1
CDK2
CDK4
MAPK14
MET
EGFR
HGF
IL6

Figure S6. Identification and validation of proteogenomic biomarkers that distinguish papillary RCC from mucinous tubular spindle cell carcinoma (MTSCC), Related to Figure 6

A. Boxplots highlight nominated pRCC type-1 mRNA markers PIGR and SOSTDC1.

B. Boxplots present the differential mRNA abundance of PIGR and SOSTDC1 across RCC subtypes among an inhouse (MCTP) and TCGA combined cohort. Asterisks mark p-value less than 0.0001 between pRCC-1, pRCC-2 and other subtypes (upper), or between pRCC-1 and all the other subtypes (lower). P-values were calculated using Wilcoxon rank sum test.

C. Volcano plot shows significant, differentially (abs(log2fc)>0.5 and p value < 0.05) regulated phosphorylation sites between missense MET mutated (n = 2) and wild type MET (n = 5) pRCC tumors. Phosphorylation sites on potential MET substrate proteins are highlighted and labeled.

D. Violin plots show distribution of gene expression fold changes between chr7 gain and no gain samples separately derived from ccRCC and non-ccRCC subgroups. P-values were calculated using Wilcoxon rank sum test.

E. Scatter plot shows the signed log2 fold change of genes's protein expression (x axis), RNA expression (y axis) derived from DE analysis comparing chromosome 7 gain with no gain samples in ccRCC (upper panel) and non-ccRCC (lower panel) subgroups. Genes on chromosome 7 are represented with solid black circles.

F. Enrichment in chromosome 7 and chromosome 17 gene sets are tested with RNA expression difference between chromosome 7 gain samples and no gain samples in ccRCC groups. P values were from GSEA.

G. Hallmark pathway enrichment analysis based on expression fold changes between chromosome 7 gain and no gain samples stratified by ccRCC and non-ccRCC subgroups with RNA expression data. Asterisk denotes p values less than 0.05 from GSEA.

H. Kinases with enriched upregulated phosphorylation (red) and downregulated phosphorylation (blue) in Chr 7 gain samples compared to Chr7 no gain samples. Chr7 kinases are colored and labeled with black.

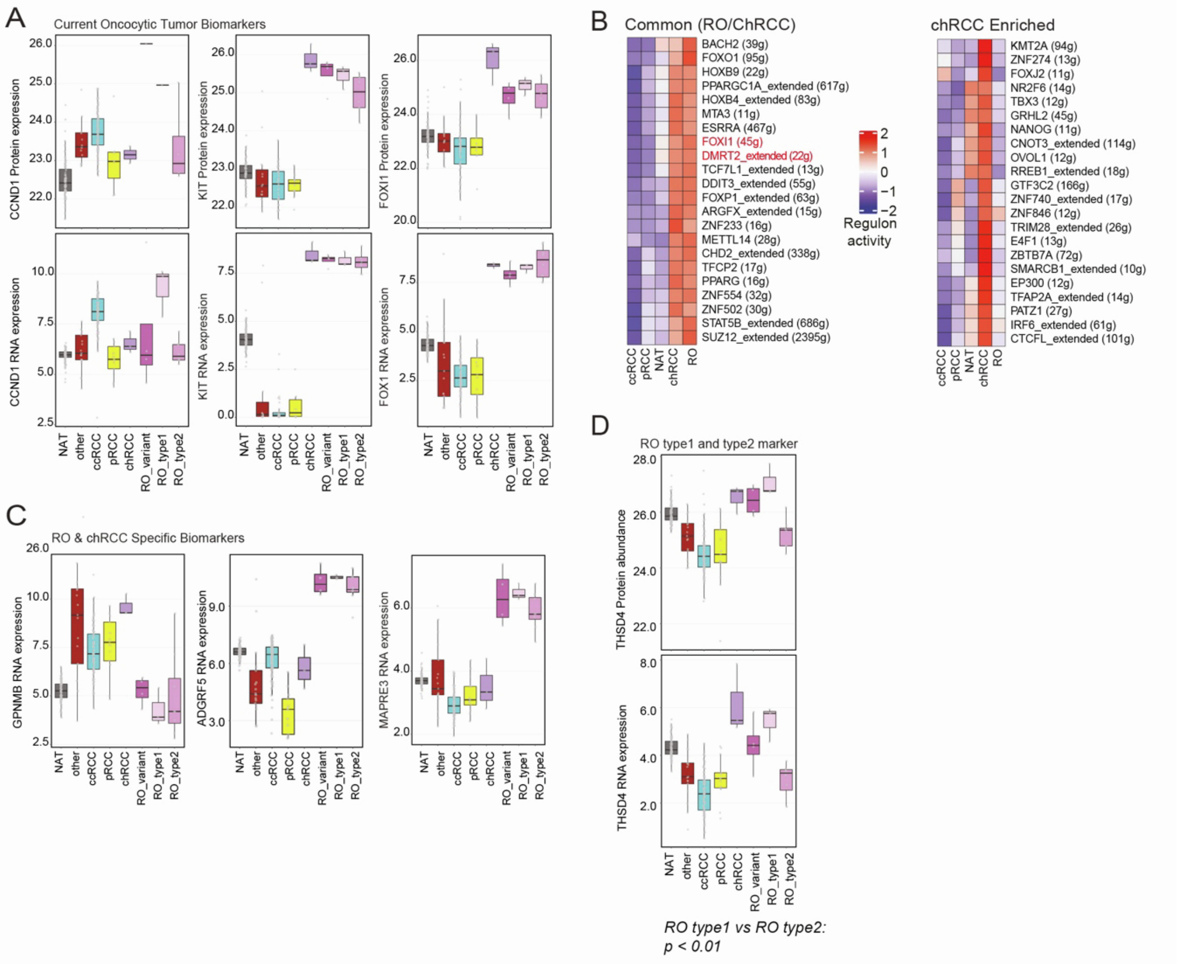

A
Current Oncocytic Tumor Biomarkers
CCND1 Protein expression
KIT Protein expression
FOXI1 Protein expression
CCND1 RNA expression
KIT RNA expression
FOXI1 RNA expression
NAT
other
ccRCC
pRCC
chRCC
RO_variant
RO_type1
RO_type2
B
Common (RO/ChRCC)
BACH2 (39g)
FOXO1 (95g)
HOXB9 (22g)
PPARGC1A_extended (617g)
HOXB4_extended (83g)
MTA3 (11g)
ESRRA (467g)
FOXI1 (45g)
DMRT2_extended (22g)
TCF7L1_extended (13g)
DDIT3_extended (55g)
FOXP1_extended (63g)
ARGFX_extended (15g)
ZNF233 (16g)
METTL14 (28g)
CHD2_extended (338g)
TFCP2 (17g)
PPARG (16g)
ZNF554 (32g)
ZNF502 (30g)
STAT5B_extended (686g)
SUZ12_extended (2395g)
Regulon activity
chRCC Enriched
KMT2A (94g)
ZNF274 (13g)
FOXJ2 (11g)
NR2F6 (14g)
TBX3 (12g)
GRHL2 (45g)
NANOG (11g)
CNOT3_extended (114g)
OVOL1 (12g)
RREB1_extended (18g)
GTF3C2 (166g)
ZNF740_extended (17g)
ZNF846 (12g)
TRIM28_extended (26g)
E4F1 (13g)
ZBTB7A (72g)
SMARCB1_extended (10g)
EP300 (12g)
TFAP2A_extended (14g)
PATZ1 (27g)
IRF6_extended (61g)
CTCFL_extended (101g)
C
RO & chRCC Specific Biomarkers
GPNMB RNA expression
ADGRF5 RNA expression
MAPRE3 RNA expression
D
RO type1 and type2 marker
THSD4 Protein abundance
THSD4 RNA expression
RO type1 vs RO type2: p < 0.01

Figure S7. Identification and validation of proteogenomic biomarkers that distinguish Oncocytomas (RO) from Chromophobe RCC (chRCC), Related to Figure 7

A. Boxplots show the protein abundance (top) and RNA expression (bottom) of known biomarkers of RO and chRCC across different kidney tumors.

B. PyScenic analysis identifies transcriptional modules commonly enriched in RO and chRCCs (left), and those specifically enriched in chRCCs (right).

C. Boxplots show the chRCC specific marker GPNMB (left) and RO specific biomarker ADGRF5 and MAPRE3 RNA expression in different tumor subtypes.

D. Boxplots show the differential protein abundance (top) and RNA expression (bottom) (between RO type-1 from RO type-2) of THSD4 across different kidney tumors. P-values were calculated using Wilcoxon rank sum test.
